# Single-Cell Phenotypic Analysis and Digital Molecular Detection Linkable by a Hydrogel Bead-Based Platform

**DOI:** 10.1021/acsabm.0c01615

**Published:** 2021-02-12

**Authors:** Yanzhe Zhu, Jing Li, Xingyu Lin, Xiao Huang, Michael R. Hoffmann

**Affiliations:** Linde+Robinson Laboratories, California Institute of Technology, Pasadena, California 91125, United States

**Keywords:** hydrogel, nanoliter, microfluidics, cell heterogeneity, digital PCR, digital LAMP, single-cell analysis

## Abstract

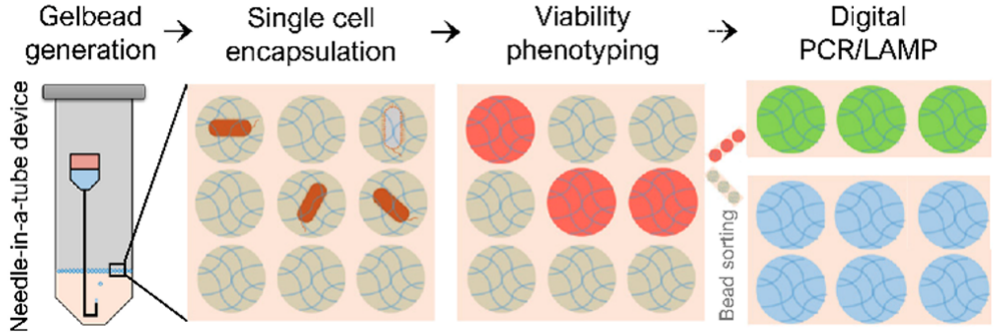

Cell heterogeneity, such as antibiotic heteroresistance and cancer cell heterogeneity, has been increasingly observed. To probe the underlying molecular mechanisms in the dynamically changing heterogeneous cells, a high throughput platform is urgently needed to establish single cell genotype-phenotype correlations. Herein, we report a platform combining single-cell viability phenotypic analysis with digital molecular detection for bacterial cells. The platform utilizes polyethylene glycol hydrogel that cross-links through a thiol-Michael addition, which is biocompatible, fast, and spontaneous. To generate uniform nanoliter-sized hydrogel beads (Gelbeads), we developed a convenient and disposable device made of needles and microcentrifuge tubes. Gelbead-based single cell viability and molecular detection assays were established. Enhanced thermal stability and uncompromised efficiency were achieved for digital polymerase chain reaction (PCR) and digital loop-mediated isothermal amplification (LAMP) within the Gelbeads. Reagent exchange for in situ PCR following viability phenotypic analyses was demonstrated. The combined analyses may address the genotypic differences between cellular subpopulations exhibiting distinct phenotypes. The platform promises unique perspectives in mechanism elucidation of environment-evolution interaction that may be extended to other cell types for medical research.

## Introduction

Microfluidic single cell techniques have enabled observations of rare genotypes or viability phenotypes within a cell population and, thus, ubiquitous cell heterogeneity.^1−3^ The phenotypic diversity exhibited by supposedly genetically identical cells boosts the population adaptability under selection pressures and, thus, raises concerns in fields spanning from clinical practice to medical research on infectious diseases and cancers,^4,5^ etc. For example, less susceptible pathogenic bacterial subpopulations originally consist of 10^–2^–10^–6^ of the overall population that can be amplified during antibiotic exposure. The subsequent increase in the resistant subpopulation may eventually lead to the failure of an antibiotic treatment.^6^ Hypotheses for the underlying molecular mechanisms, involving the stochasticity of genetic mutation, gene expression, and protein regulation,^5,7,8^ however, remain hard to test in dynamically changing cell subpopulations, partly because of the absence of appropriate single cell experimental techniques.^9^ For example, a single-cell analysis technique that evaluates both the viability phenotype and the presence of certain genes may help better elucidate the role of the genes play in the fitness of individual cells. The need to better understand cell heterogeneity motivates the development of new techniques that link the single-cell viability phenotype with its in situ molecular information.

As an emerging class of technologies, water-in-oil droplet-based microfluidic platforms have been well developed for high-throughput phenotypic and molecular analyses at single cell or single molecule resolution.^2,10−12^ Nonetheless, because of the rare and transient nature of cell heterogeneity events, population-averaged molecular analyses would most likely fail to directly explain the characterized phenotypes, even if all analyses are conducted at single cell or molecular resolution.^6,13^ Incorporation of a cross-linked hydrogel network into the aqueous phase theoretically provides a droplet-based platform with additional robustness by allowing reagent exchange.^14^ This strategy has been explored for a range of hydrogel materials and cross-linking chemistry, including cooling-induced formation of agarose beads for digital droplet polymerase chain reactions (ddPCR),^15^ ionic cross-linking of alginate beads for cell encapsulation and DNA extraction,^16,17^ and UV-initiated polyethylene glycol (PEG) beads for cell encapsulation.^18^ Various platforms have demonstrated to be effective for either phenotyping or molecular analysis, while the material or initiation method would be intrinsically incompatible with the combination of both. For example, elevated temperature during agarose microsphere generation and cell encapsulation may complicate the droplet generation process and increase mutation rates,^19^ UV radiation may affect the phenotype and genotype of encapsulated cells,^20^ and finally, alginate is a well-known PCR inhibitor.^21^ PEG cross-linked by a thiol-Michael addition reaction between the bioinert acrylate and thiol groups has been attempted in bulk analyses and is among the most promising solutions,^22,23^ but it is yet to be developed for our specific purpose. The main obstacle may lie in the fast and spontaneous gelation, which would be detrimental to traditional expensive microfluidic droplet generation approaches.

**Figure 1:**
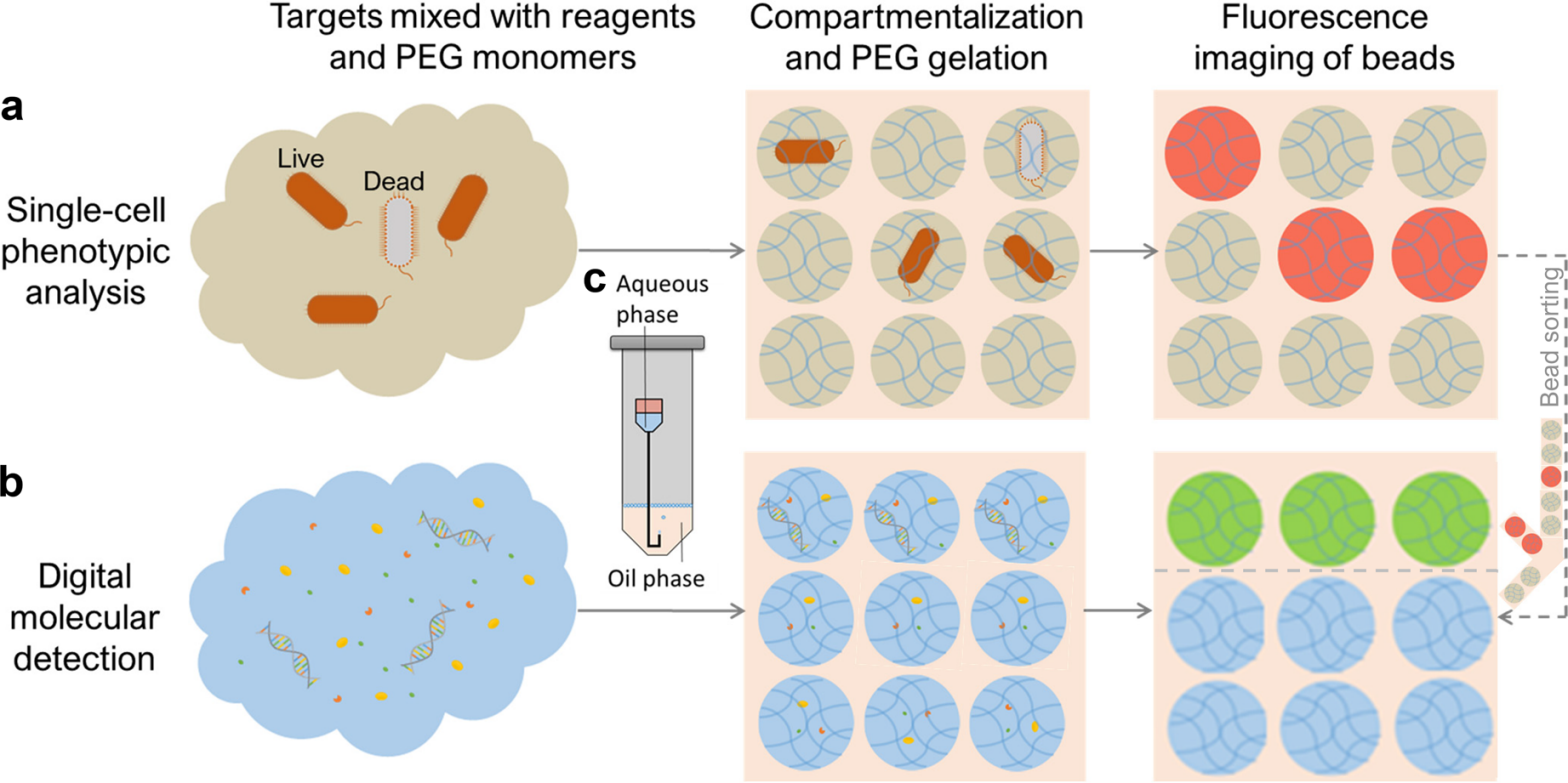
Schematic of this study. Hydrogel bead (Gelbeads)-based cell analysis platform was developed for (a) single-cell phenotypic analysis and (b) digital molecular detection including PCR and LAMP. Compartmentalization was realized by (c) a disposable centrifugal droplet generation device. The dashed-line arrow indicates that the immediate potential of linking cell phenotype with in situ DNA/RNA characterization at single-cell resolution.

Herein, we report a novel PEG hydrogel bead-based platform for linking single-cell phenotypic analysis and in situ molecular detection (Figure 1a and b). To solve the challenge posed by the fast thiol-Michael addition gelation chemistry, we developed a disposable centrifugal device for droplet generation (Figure 1c). With generated nanoliter-sized droplets, which are further spontaneously cross-linked into PEG hydrogel beads (Gelbeads), we established single cell encapsulation and effective viability phenotyping within 4 h for the tested bacteria. Gelbead-based assays were also developed for nucleic acid amplification detections, including PCR and loop-mediated isothermal amplification (LAMP). Compared to droplet digital PCR and LAMP (ddPCR and ddLAMP), Gelbead-based digital PCR and LAMP (gdPCR and gdLAMP) are shown to have enhanced thermal stabilities and uncompromised amplification efficiencies. Phase transfer and reagent infusions for in situ PCR following Gelbead-based viability phenotyping were successfully conducted. The Gelbead platform reported here has the potential to extend to study of other types of cells and promises unique capabilities for investigation of cell heterogeneity and, thus, have broad interest in many biological research fields.

## Results and Discussion

### Development of the Disposable Droplet Generation Device

**Figure 2:**
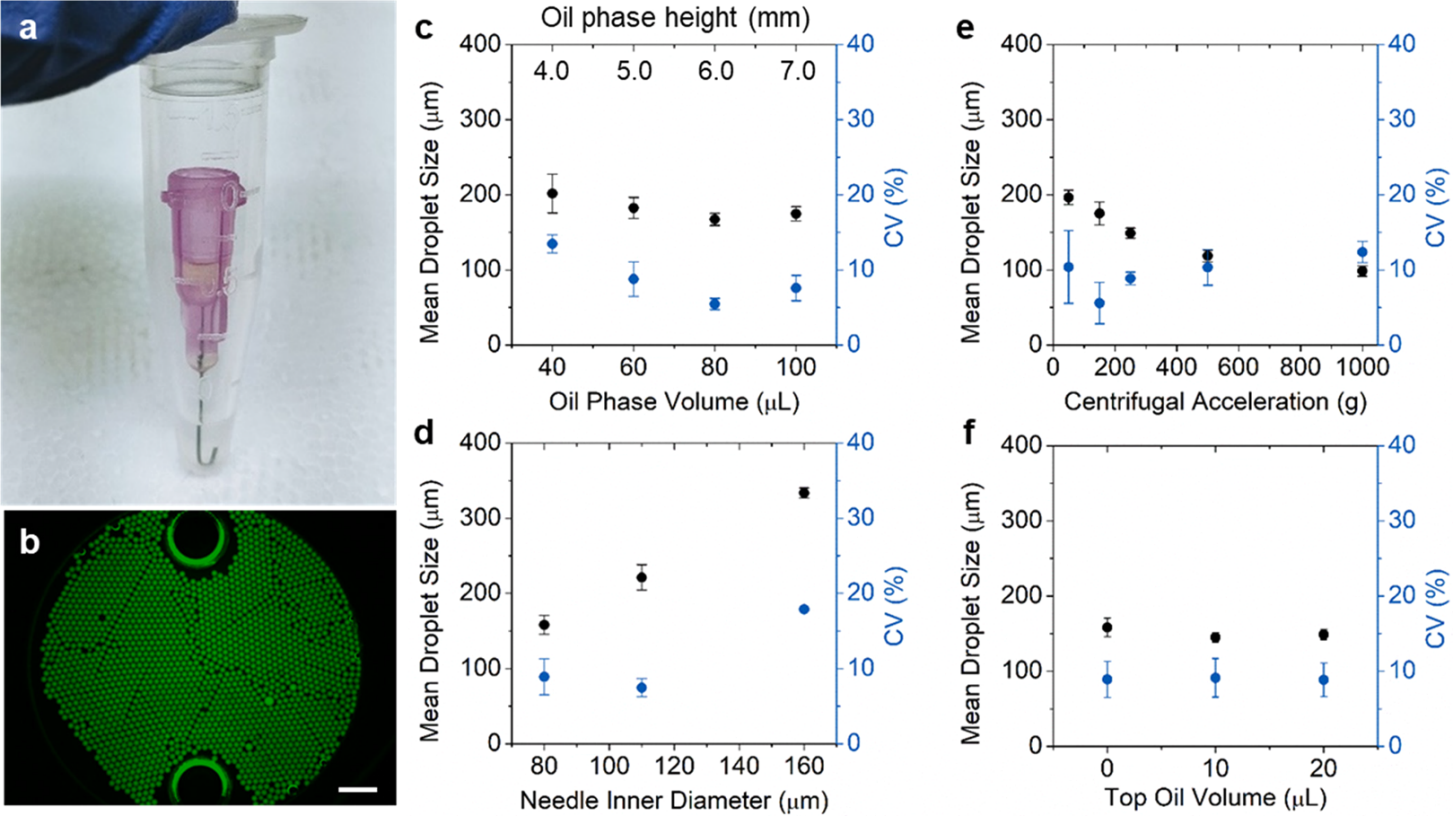
Development and evaluation of NeaT droplet generation. (a) Device setup consisting of a 1.5 mL microcentrifuge tube holding the oil phase and a needle with bent tip holding the aqueous reaction mixture in the Luer-lock. (b) Representative fluorescence microscope image of generated droplets extracted into a viewing chamber. The two large bright circles are ports on the viewing chamber for liquid loading Scale bar, 1 mm. (c–f) Mean droplet size (black circles) and CV (blue circles) of droplets produced under varying parameters including (c) oil phase volume, (d) needle inner diameter, (e) centrifugal acceleration, and (f) oil volume added to the Luer-lock. Error bars represent standard deviation from independent triplicates.

Microfluidic-based droplet generation methods generally require special fabrication facilities to generate sub-100-μm channels. They also require syringe pump-driven T-junctions fabricated by photolithography and centrifugally driven laboratories-on-a-disc fabricated by micro milling and hot embossing.^24,25^ These traditional methods are not compatible with Gelbead generation due to fast clogging imposed by the thiol-Michael addition chemistry. Bulk PEG cross-linking experiments show that the time frame for droplet generation before gelation is as short as 8.5 min at a hydrogel concentration of 7.5 w/v% (Supplementary Note 1, Table S1). To easily generate Gelbeads within minutes without clogging expensive microfluidic equipment, we designed a needle-in-a-tube (NeaT) device, which is a disposable droplet generation device using affordable commercial components (Figure 2a). The device utilizes a dispensing blunt needle with a bent tip. The bent-tipped needle is then set into a 1.5 mL microcentrifuge tube with oil to establish the physics for centrifugal droplet generation (Supplementary Note 2, Movie S1). With centrifugal acceleration, the aqueous phase is forced into the fluorinated oil phase by the elevated pressure difference between the reservoir surface and the narrow inlet. The fluorinated oil phase with a higher density pinches off the aqueous droplets, which then float to the air–oil interface. Ten needles were randomly selected after droplet generation, and the length of the bent tip was measured to be 1.8 ± 0.1 mm, indicating that manual fabrication can be reproducible.

Standard 20 μL of LAMP mix with unquenched calcein was dispersed in fluorinated oil (online methods) and characterized using a fluorescence microscope to study the droplet generation performance of the device (Figure 2b). The average droplet size was tunable from 99 to 334 μm, and the coefficient of variance (CV) was minimized to 5%, by varying the oil phase volume, centrifugal acceleration, and the needle gauge as shown in Figure 2c–f. Smaller droplets with slightly larger size distributions (Figure 2e) were produced by increasing the centrifugal acceleration, which provided a greater pressure difference to drive the aqueous phase inflow. The larger CV in Figure 2e was likely due to the unstable flow during initial acceleration, which can be alleviated by adding more oil (Figure 2c) to reduce the oil phase height variation and limit the amount of aqueous phase inlet during acceleration. Among all tested conditions, the optimal CV was found to be a combination of 34 Ga needles, 80 μL of oil phase, and 150*g* centrifugation run for 5 min, and droplets were produced at an average diameter of 175 μm in 5 min with minor trial-to-trial difference. The droplet generation rate is thus estimated to be on the order of 10^3^ droplets per min. This approach is comparable to other microfluidic methods such as centrifugal lab-on-a-disk^24^ and polymer-tube micronozzles (Supplementary Note 3).^26^ The average diameter of 175 μm is a reasonable size for this study, since droplets with 100–200 μm diameters are commonly used for cell analyses.^24,27^ For droplets with this size, each standard 20 μL reaction could theoretically produce more than 7000 droplets of 2.8 nL in volume. On the basis of this calculated compartmentalization, the dynamic range is theoretically predicted from 0.5 to 3 × 10^3^ target copies or cells per microliter with a detection limit of 0.1 copies or cells per microliter.^28^ Through COMSOL simulation of the two-phase flow at the needle tip (Figure S1, Supplementary Note 4), it was found that the bending angle of the needle has limited impact on the size of generated droplets. This provides a theoretical basis of the consistent performance of NeaT droplet generation from trial to trial. It should be noted that droplet size increases linearly with interfacial tension, suggesting that measures to lower interfacial tension may help achieve decreased droplet size. When extending the NeaT droplet generation to other applications, optimization may be needed for a new system that has a different aqueous phase viscosity and interfacial tension.

### Gelbead Generation and Thermal Stability Characterization

**Figure 3:**
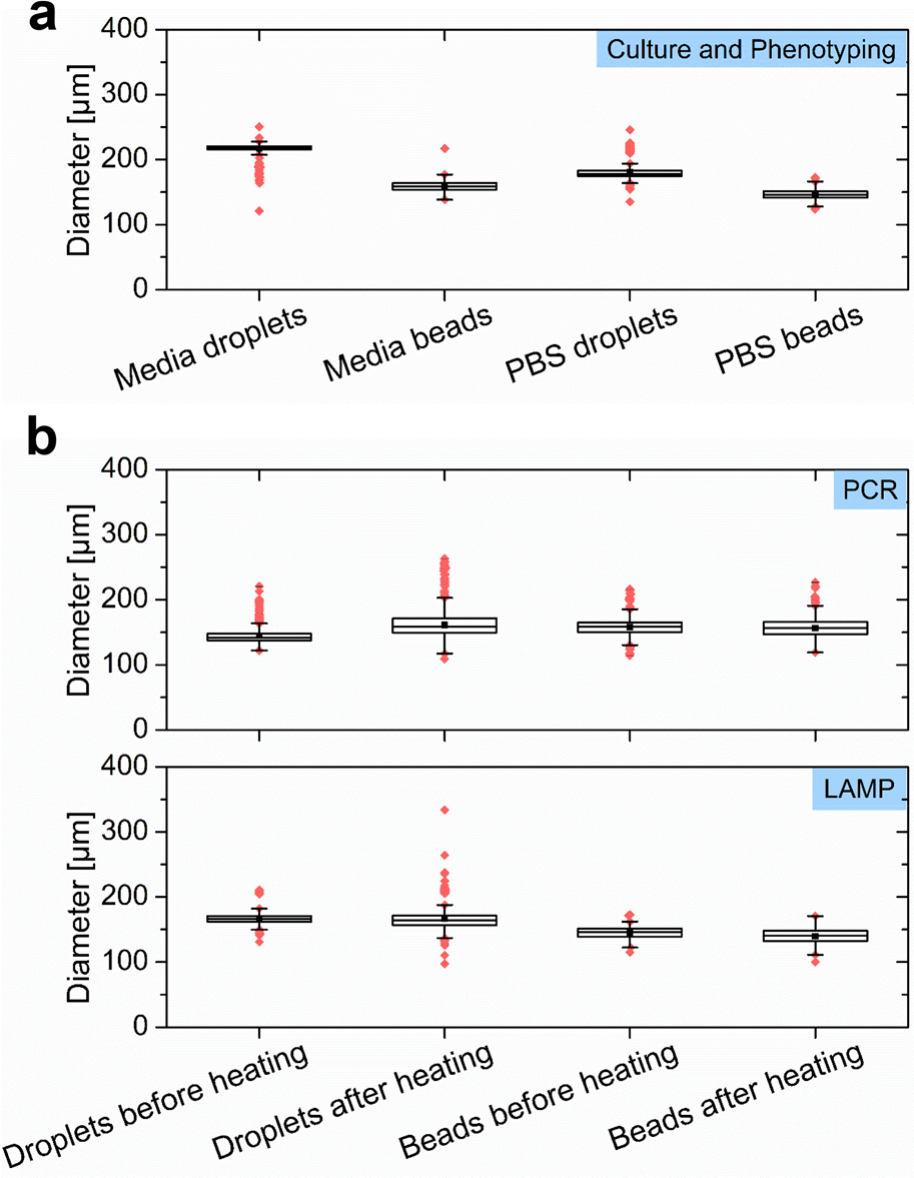
Size characterization of droplets and Gelbeads. The size distribution of droplets and Gelbeads (a) generated in reaction matrices, including PCR mix, LAMP mix, and culture media mix, and (b) before and after heating program designated for PCR and LAMP. The line inside each box represents the mean diameter; the lower and upper edges of each box, respectively, represent 25% and 75% percentiles; the vertical bars below and above each box, respectively, indicate 90th and 10th percentiles. The lower and upper red dots stand for outliers, which are points located outside the whiskers.

The Gelbead and droplet generation performances were assessed using various reaction matrices including culture media, PCR mix, and LAMP mix, under the optimized condition reported in the previous section (Figure 3a). For these reaction matrices, we did not observe any noticeable difference in the time required for complete emulsification of 20 μL aqueous phase, indicating a consistent droplet generation rate across matrices. The average diameter of generated Gelbeads was found to range from 145 to 217 μm with a CV from 3.6% to 7.6%. The observed variations were likely due to viscosity differences and interfacial property changes in different reaction matrices. It should be noted that the culture media alone was not able to sustain as droplets or Gelbeads in the fluorinated oil using a 5% FluoroSurfactant. Bovine serum albumin (BSA), a protein commonly used as an additive to protect essential molecules (fatty acids, amino acids, etc.) in culture media,^29^ was added to the aqueous phase as an additional surfactant to modify interfacial properties and thus prevent the droplet merging. For the PCR reaction matrix, the generated Gelbeads had a larger CV than droplets. We assume that the presence of PEG hydrogel may have disturbed the surfactant-stabilized aqueous-oil interface, by inducing interfacial adsorption of additional charged species such as thiolate, magnesium ions, etc. In summary, the observed sizes and CVs of droplets and Gelbeads were considered acceptable for our assays. In general, our simple generation device fulfills the requirements for Gelbead generation. The generation device may be used for applications for which a simple yet powerful compartmentalization method is needed.

The effect of PEG cross-linking on stabilizing the aqueous-in-oil compartments was evaluated. Thermodynamic instability of water-in oil droplets may impair the reliability of amplification processes such as PCR and LAMP that require extensive heating.^30^ Heating accelerates droplet merging and evaporation, which would affect the fluorescence reading by modifying concentrations of targets and reagents (e.g., salts and fluorescent dyes). In this context, the compartmental heat stability manifests through the dispersion of droplet/Gelbead sizes before and after heating. The sizes were investigated for droplets and Gelbeads before and after common heating protocols respectively for PCR and LAMP (online methods, Figure 3b). Compared to those before heating, droplets that had undergone PCR and LAMP heating increased in their CVs by 6.2% and 3.5%, respectively. In addition, the heating resulted in a noticeably larger population with both larger and smaller outlier sizes implying that extensive merging and evaporation had occurred. Following the same heating protocol as for the droplets, the Gelbeads exhibited much less of a change in size distribution (CV increased by 1.9% for PCR and 1.6% for LAMP). However, the average Gelbead diameter decreased slightly. These results indicate that the stabilization effect achieved by cross-linked PEG was mainly by preventing merging of compartments, and lessen the extent of aqueous evaporation. The effect of mild aqueous evaporation in Gelbeads can be compensated by optimization of assay recipes. Gelbeads used for the LAMP procedure had a significant improvement in thermal stability because of PEG cross-linking than for the PCR procedure. We assume that, in the case of the PCR recipe, the combination of SuperMix and the oil phase from BioRad were chemically well-optimized for interfacial stability, leaving limited room for improvement. This result therefore indicates that, other than modifying the surfactant composition or increasing surfactant concentration, hydrogel cross-linking could be an alternative strategy for maintaining the emulsion. Our results demonstrate that Gelbeads are a reliable platform for standalone heated digital analysis in terms of enhanced individual compartment integrity.

### Gelbeads for Cell Viability Phenotyping

**Figure 4:**
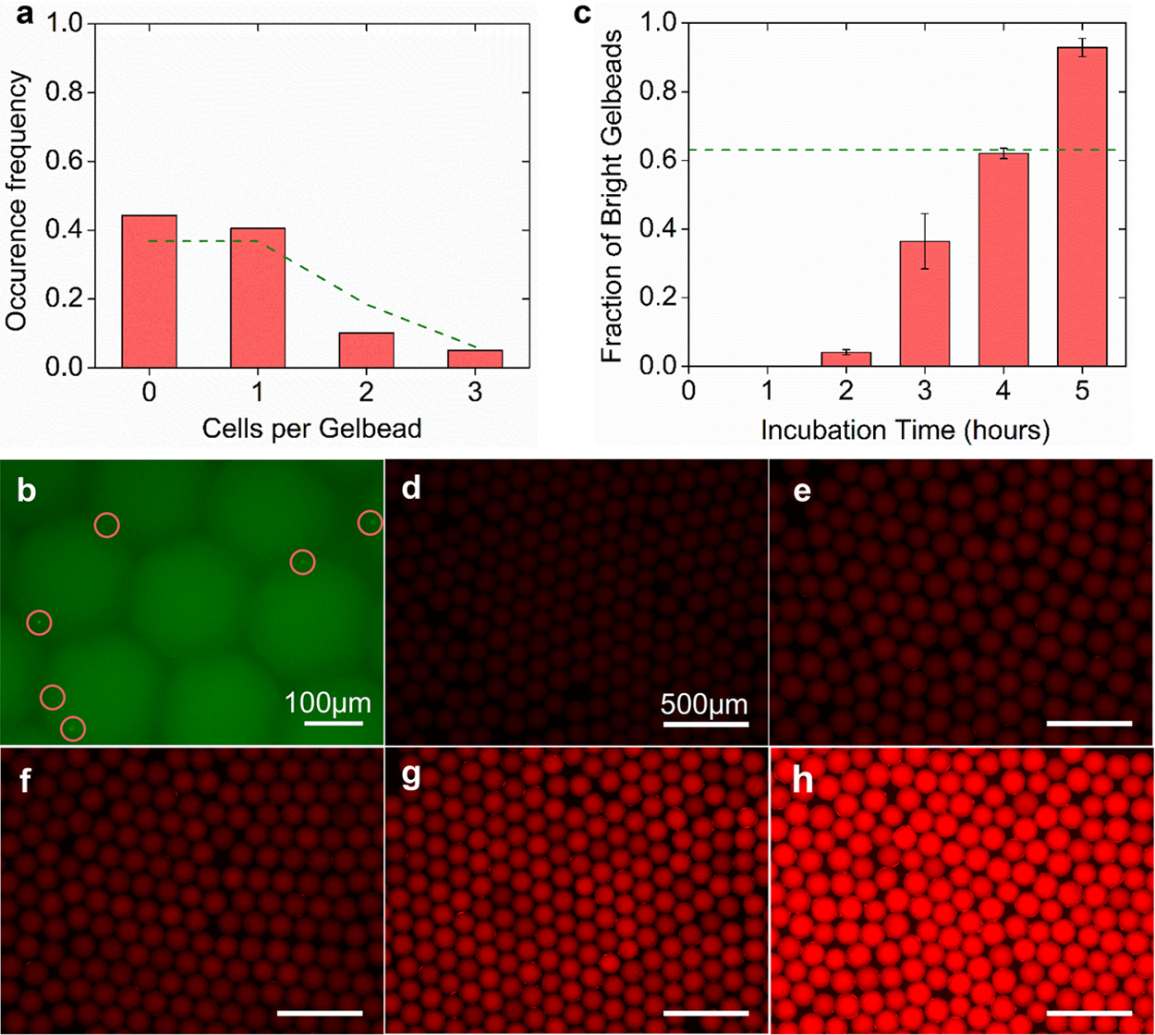
Single-cell encapsulation validation and viability phenotyping performance in Gelbeads. (a) Number of cells encapsulated in each Gelbead counted and represented by occurrence frequency. The dashed lines represent theoretical values based on Poisson distribution. (b) Example fluorescence image of encapsulated *Salmonella* typhimurium GFP cells (circled) for counting. Scale bar: 100 μm. (c) Observed fraction of bright Gelbeads with varying incubation time, with the dashed line representing 63% as Poisson distribution predicted based on the input cell concentration. Error bars represent standard deviation from independent triplicates. (d–h) Example images of Gelbeads containing *Salmonella* typhi at the same input concentration incubated for 0, 2, 3, 4, 5 h. Scale bars: 500 μm.

The distribution of cells in Gelbeads was experimentally characterized using *Salmonella* Typhimurium with green fluorescent protein (*S.* Typhimurium GFP). To obtain the highest single cell encapsulation efficiency, the cells were diluted to an average of 1 cell per Gelbead for droplet generation. The number of cells encapsulated in each Gelbead was counted (Figure 4b). At this cell concentration, theoretically, 34% of the compartments were occupied by single cells, which was the maximum following a Poisson distribution, 29% of the compartments encapsulated more than 1 cell, and 37% of the compartments contained no cells. As shown in Figure 4a, the observed number of encapsulated cells was close to the theoretical distribution. The number of Gelbeads containing high cell numbers was slightly less than predicted, likely because some cells were located out of focus when imaged in spherical compartments at a high microscope objective. The possibility that this observation was caused by cell sedimentation was found to be negligible, according to the numerical simulations (Figure S2, Supplementary Note 5). In the numerical experiments, under the low centrifugal acceleration used in droplet generation, the drag force and centrifugal force exerted on the cell particles did not induce a significant change in compartmentalization pattern. Since high throughput detection of stained cells within spherical compartments droplets or Gelbeads was challenging for fluorescence microscope imaging, we then employed cell metabolism indicator dye in Gelbead viability phenotyping experiments.

Gelbead-based viability phenotyping performance was investigated by coincubation of alamarBlue and *Salmonella* typhi in the culture media. As a resazurin-based dye used in bulk phenotyping assays of a wide range of cell lines, alamarBlue can be reduced by actively metabolizing cells into resorufin, whose bright red fluorescence can stain the whole compartment for visualization.^31^ The fluorescence of Gelbeads was monitored during the incubation for up to 4 h (Figure 4d–h). It was observed that Gelbeads appeared to be much brighter than the droplets were before incubation (Figure S3); this was possibly due to additional reduction of resazurin by the thiol group.^32^ We suppose that the interference by thiol groups would not affect the phenotyping results since the monomers were rigorously mixed and evenly distributed into Gelbeads. Gelbeads containing live cells would exhibit even brighter fluorescence in the presence of sufficient AlamarBlue.

The quantitative performance of viability phenotyping with Gelbeads was assessed by analysis of observed fractions of bright fluorescent Gelbeads (see online methods and Figure S4 for thresholding) compared to the theoretical value, as shown in Figure 4c. According to theoretical estimation, 63% of Gelbeads were supposed to contain greater than or equal to 1 cell and thus to be bright. The observed positive fraction of 62.0 ± 1.5% after 4 h of incubation matched well with the theoretical value of 63%. It was also noticed that, after 3 h of incubation, the positive Gelbead fraction was 36.4 ± 8.1%, which corresponds well with the theoretical fraction of Gelbeads (26%) encapsulating more than 1 cell. Based on the linear response of alamarBlue to the number of cells within the compartment,^33^ our results reasonably indicate that effective single cell phenotyping in Gelbeads is achievable within 4 h. However, 5 h incubation lead to overly bright fluorescence and 92.9 ± 2.7% bright Gelbeads, which was likely attributed to excessive incubation and the diffusion of metabolized fluorescent resorufin across the aqueous–oil interfacial barrier. We note that interfacial leakage of the metabolized dye molecules might also have reduced the fluorescence intensity difference between bright and dark Gelbeads for incubation below 5 h. These observations indicate that the optimization of incubation time is a race between cross-talking and cell proliferation. The incorporation of PEG hydrogel into aqueous phase likely has already enhanced the retention of the resorufin-based dye due to its preferred partition in the PEG layer, as illustrated in the dropicle system developed by Wu et al.^34^ The leakage can be further reduced by changing to another specific type of oil phase, indicator dye molecule, or surfactant.^35^ Considering the intrinsic difference in proliferation rate between bacterial species, the observed incubation time for distinction of positive and negative compartments was comparable to the results by Lyu et al., who achieved *Escherichia coli* (*E. coli*) phenotyping with alamarBlue in 85 pL droplets with a 2 h incubation.^2^

In summary, Gelbeads synthesized in this study could act as a platform for characterizing phenotypic cell heterogeneity if coencapsulated with antibiotics or drugs. The cell viability detection strategy demonstrated with Gelbeads has been proved to apply well to a wide range of cells in bulk assays and droplet microfluidics.^2,31,33^ We note that here the single cell encapsulated Gelbeads were at the highest yield under Poisson distribution so that they theoretically comprised the majority (59%) of the bright Gelbeads in the current setup. The input cell concentration can be diluted to increase the single cell encapsulation among positive Gelbeads to above 99%.^36^ Or the single cell encapsulated Gelbeads could potentially be sorted out through on-chip imaging of the Gelbeads to count the fluorescently labeled cells.^37^

### Gelbead Digital PCR (gdPCR)

**Figure 5:**
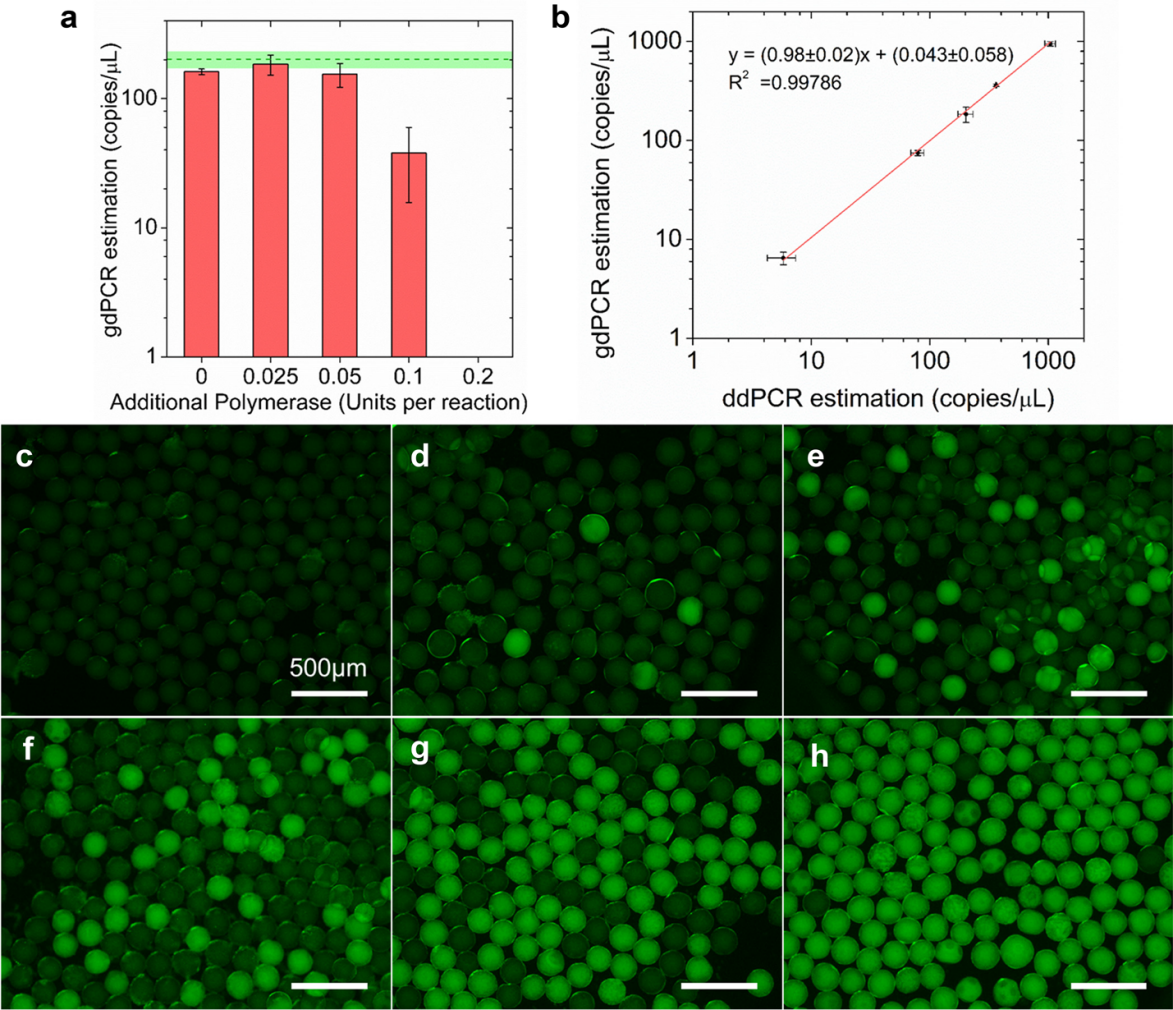
Optimization and performance of gdPCR. (a) Concentration estimations of gdPCR assays for a fixed input *S.* typhi DNA concentration (200 copies/μL) with varying concentrations of additional polymerase. The green dashed line and the green area represent mean concentration estimation with standard deviation of ddPCR assays from independent triplicates. (b) With the optimized additional polymerase concentration (0.025 Units per reaction), the correlation between gdPCR and ddPCR estimation for serial diluted target templates. Error bars represent standard deviations from independent triplicates. (c–h) Example gdPCR fluorescent images for no DNA input, and with 24000, 1500, 600, 300, 100 times dilution of harvested *S.* typhi DNA. Scale bars: 500 μm.

To establish a reliable gdPCR assay, we investigated the amplification efficiency of gdPCR compared to digital PCR performed in droplets generated from a commercial recipe (represented as ddPCR, hereinafter) with DNA extracted from cultured *Salmonella* typhi (*S.* typhi). Previous studies of hydrogels and PCR mostly utilized polyacrylamide in the form of either a bulk phase hydrogel membrane as a quasi-digital PCR platform^38^ or using hydrogel beads as a substrate for surface coating of primers,^39,40^ which is an approach different from our concept. Novak et al. demonstrated ∼100% efficiency for multiplex PCR amplification of single cells inside agarose droplets, which are in a melted state under temperatures during PCR.^41^ To the best of our knowledge, performing PCR inside cross-linked hydrogel beads has not been reported to date. Even in bulk membrane form, only 80% amplification efficiency was observed, which may be partially attributed to template damage by free radicals as suggested.^38^ In this study, a similar drop in amplification efficiency was observed in the Gelbeads compared to that in droplets (Figure 5a), even though the Michael addition chemistry between acrylate and thiol used in this study does not involve free radical formation. In this case, cross-linked hydrogel network may be responsible for the observed inhibition by limiting the diffusion of functional components such as ions, nucleic acids, and proteins, where the extent of the limitation relates to the size and charge of the component.^42,43^

From effective diffusivity modeling (Figure S5), we reasoned that the most affected functional component might be DNA polymerase, which is the relatively large protein (∼6 nm) responsible for building amplicons. For a fixed template concentration of 200 copies/μL estimated by ddPCR, gdPCR assay performance was assessed with additional OneTaq polymerase supplied at varying concentrations of 0.025, 0.05, 0.1, 0.2 units per reaction, as shown in Figure 5a. Results showed that additional 0.025 Unit per reaction, 5% of the recommended OneTaq polymerase concentration per reaction, boosted the amplification efficiency the most. OneTaq polymerase concentrations supplied more or less than that showed inhibition to amplification efficiency, and gdPCR assay with additional 0.2 unit per reaction was shown to be completely inhibited. We speculate that the observed trend was mainly due to the commercial SuperMix buffer conditions not optimized for the supplied OneTaq polymerase. While some additional polymerase compensated the reduced diffusivity of the SuperMix polymerase in hydrogel, the excess additional OneTaq polymerase caused failure in detection. We suspect that severe nonspecific amplifications might have occurred so that the products could not induce hydrolysis of the sequence-specific TaqMan probe.

With the optimized additional polymerase, gdPCR assays for serially diluted DNA with concentrations ranging from 2.5 to 600 copies/μL were then performed; typical images are shown in Figure 5c–h (Supplementary Note 6). The image analysis results demonstrated that the amplification efficiency of gdPCR was comparable (*k* = 0.98 ± 0.02, *R*^2^ = 0.9979) to that of ddPCR with the recipe adjustment (Figure 5b). The quantification results also correlated well with input DNA concentration (Figure S6a). It should be noted that the cross-linking inhibition effect eliminated in this case was for a 131 bp target gene,^44^ a typical size for detection of specific bacteria. Further optimization in polymerase or Supermix concentration would be required for other applications if a larger DNA fragment is targeted.

### Gelbeads Digital LAMP (gdLAMP)

**Figure 6:**
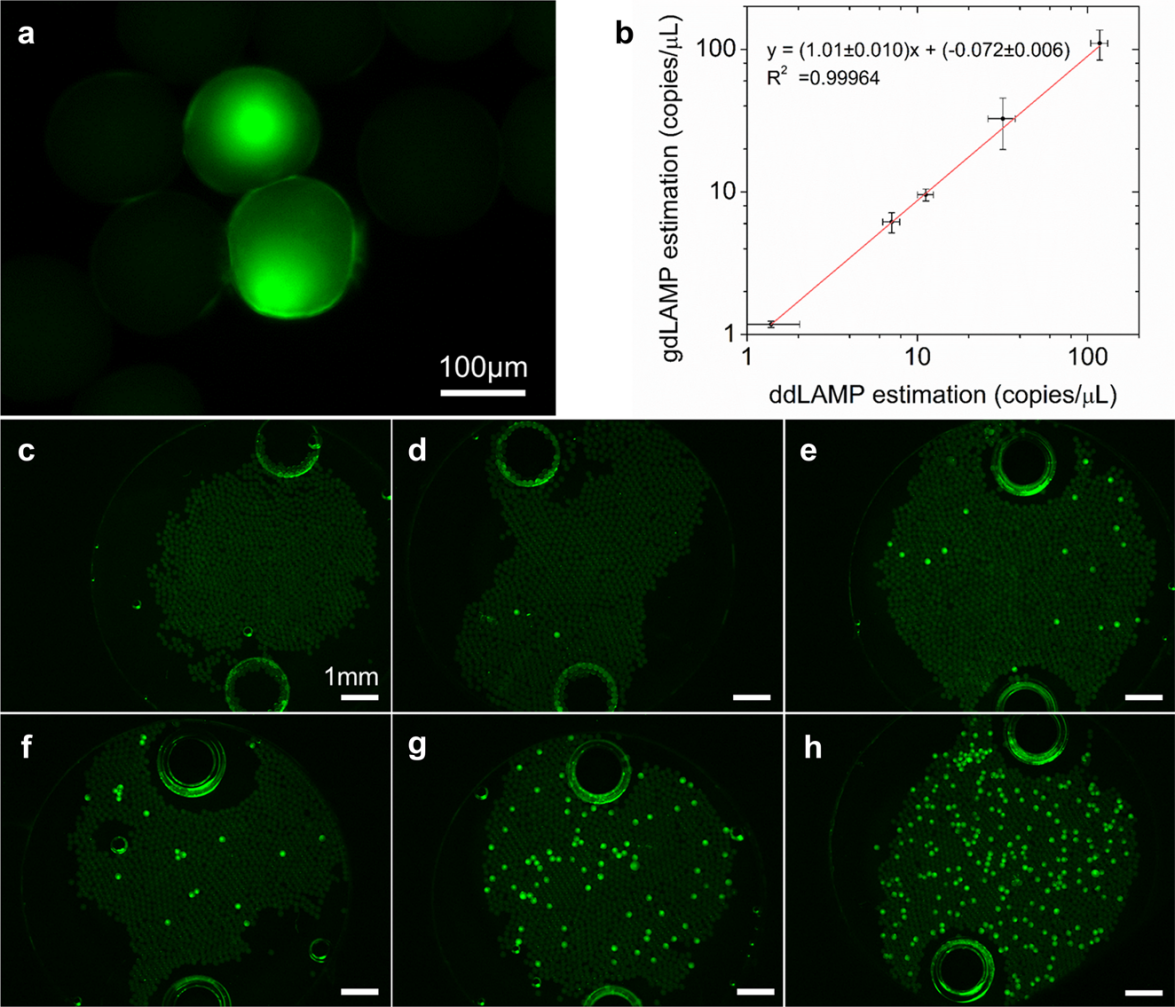
Performance of gdLAMP. (a) Connection of two positive Gelbeads after the gdLAMP assay. Scale bar: 100 μm. (b) Correlation between concentration estimations of gdLAMP and ddLAMP assays for serial diluted target templates. Error bars represent standard deviation from independent triplicates. (c–h) Example gdLAMP fluorescent images for no DNA input and with 200, 100, 50, 20, 5 times dilution of harvested *S.* typhi DNA. The two large bright circles on each image are ports on the viewing chamber for liquid loading. Scale bars: 1 mm.

Gelbead-based molecular analysis with LAMP was also investigated. LAMP has been an attractive emerging platform for molecular detection since it eliminates the need for thermocycling by utilizing a combination of 4 or 6 primers to achieve fast and specific detection.^45^ The heating protocol of LAMP was fairly mild; however, severe Gelbead aggregation occurred for samples with target DNA but not for no-template controls (Figure S7) in preliminary experiments. This was supposedly due to the fact that LAMP produces a much larger amount of amplification products than PCR.^45^ The negatively charged amplified DNA may have affected interfacial tension when adsorbed to the interface. Aggregated Gelbeads showed apparent crosstalking, which rendered the assay invalid since the compartment independence assumption required for Poisson statistics was contradicted. The problem was relieved by adding 1.5 mg/mL BSA, a common real-time PCR additive, to prevent surface adsorption. However, it was still observed that positive Gelbeads tended to stick next to each other (Figure 6a). The observed radiative patterns in Gelbeads manifested the differential diffusivity of amplification products of varying size in cross-linked hydrogel network. A similar radiative pattern was observed by Huang et al. in LAMP performed in a hydrogel membrane.^23^ In our case, neither of the two radiative centers were at the connected interface, indicating that the stickiness may not have led to false positive Gelbeads within the time frame tested. The connection of positive Gelbeads was most likely the result of a change in interfacial tension caused by large amount of the negatively charged DNA produced during amplification. Further cross-linking breaking through the oil barrier would only occur when the positive Gelbeads encounter each other. In summary, the connected interface should not affect the quantification results.

The gdLAMP quantifications for no-template control and serial diluted *S.* typhi DNA ranging from 300 to 1.2 × 10^4^ copies/μL were then verified. Example images are shown in Figure 6c–h. The image analysis results demonstrated that the amplification efficiency of gdLAMP was similar (*k* = 1.01 ± 0.01, *R*^2^ = 0.9996) to that of ddLAMP (Figure 6b). However, both ddLAMP and gdLAMP gave concentration estimations lower than input DNA concentration (Figure S6b). Further increases in the amplification efficiency would likely require an improved primer design, which is out of the scope of this study. In summary, the results confirmed our hypothesis that the stickiness of positive Gelbeads do not considerably affect gdLAMP quantification, and demonstrated that the hydrogel network had a negligible inhibition effect on the digital LAMP assays that were performed.

### Reagent Exchange for in Situ PCR Following Viability Phenotyping

With reagent exchange enabled by the cross-linked network of hydrogel, our Gelbead-based platform has the immediate potential to link phenotyping and in situ molecular detection for single cells. Previous research has demonstrated that multiplex PCR reaction could be highly efficient after equilibration with PCR reagents and re-emulsification of the agarose beads. The agarose beads have a relatively loose network with pore size at ∼130 nm,^41^ compared to our Gelbeads with pore size at 27 nm. Here we develop the reagent exchange protocol to demonstrate the feasibility to combine Gelbead-based *S.* typhi viability phenotyping and in situ PCR for *S.* typhi-specific STY0201 gene using assays established in previous sections. The key to linking the analyses lies in the effective phase transfer and reagent infusion. After single cell phenotyping (Figure S8a), successful phase transfer was conducted (Figure S8b–h) with little loss of Gelbeads and minimal emulsified aqueous reagent leftover. The challenge posed by the possibly denser interfacial hydrogel network, which might have hindered the inward diffusion of essential PCR macromolecules, was overcome by a freeze–thaw treatment (Supplementary Note 7). Resuspended Gelbeads remained intact after subsequent PCR and allowed for fluorescence analysis (Figure S8i). The fluorescence intensity profiles for randomly analyzed 5 positive and 5 negative Gelbeads were statistically distinguishable, with 40% significant difference (*p* < 0.001, one-way ANOVA) in mean fluorescence intensity (Figure S9, Supplementary Note 8). The results suggest that, after phase transfer protocol, the PCR reagents were successfully infused into Gelbeads for target amplification to proceed inside. It is, thus, feasible to combine single-cell phenotype–genotype analysis using our developed platform. However, to generate new knowledge and deepen our phenotype–genotype understanding, such as probing the correlation of an antibiotic-resistant phenotype and the presence or absence of a gene that might be essential to resistance, bead sorting that separates varying phenotypes prior to molecular analysis would be indispensable. Commercialized bead sorting facilities can be found, and we are also developing a portable and affordable bead sorting device,^46^ which is out of the scope of this manuscript.

## Conclusion

The developed Gelbeads platform promises a robust analysis tool that could potentially link single-cell phenotypic analysis with in situ molecular detection. Besides the advantages presented, we acknowledge the following limitations. First, the dynamic range in our study was restricted by the size of the compartments generated by our device. Further reductions in size would result in larger size variations, and the surfactant may have to be changed or adjusted if higher uniformity is required. Second, given the use of fluorescence microscopic imaging of the compartments inside a viewing chamber, the Gelbead imaging approach employed could probe only a limited viewing area, and the resolution could be affected by the focus. The fluorescence characterization may be further improved by interrogating a single Gelbead with fluorescence-activated bead sorter or in double emulsion with flow cytometry.

In this work, a disposable centrifugal device was developed for Gelbead generation using highly biocompatible PEG monomers spontaneously cross-linked with no free-radical, UV-induced or heat-induced initiation. Our design allows for easy adoption of droplet microfluidics without expensive and complicated equipment, which could be useful for applications other than Gelbeads generation. In addition to single cell phenotyping, the Gelbeads showed enhanced thermal stability coupled with high amplification efficiency for dPCR and dLAMP. Widely available qPCR and LAMP assays can therefore be easily transferred into digital assays by the Gelbead approach. The unique structural stability of the hydrogel network allows for easy manipulation of the Gelbeads that may have many possibilities for other upstream and downstream analyses. The reagent exchange protocol was developed for in situ PCR following Gelbead single cell viability phenotyping to demonstrate the feasibility of combining multiple analyses with Gelbeads. The Gelbead platform will be further developed for fluorescence-based Gelbead sorting and downstream sequencing, etc. Since the cells are encapsulated into individual compartments, the viability phenotype can be observed independent of intercellular collaboration, which is common for bacterial cells under pressure.^47,48^ After the Gelbeads containing cells of similar phenotype are sorted together, the differential genotypic trait may then be directly analyzed in situ with high throughput. We envision that the potential of our Gelbeads platform in generating genetic and gene expression data with phenotyped single cells will help narrow the genotype-phenotype knowledge gap and thus offer exciting new insights in cell heterogeneity studies.

## Materials and Methods

### PEG Cross-linking and Characterizations

PEG hydrogel monomers included 4-arm PEG-acrylate [molecular weight (MW) of 10 000, Laysan Bio, Arab, AL, USA] and thiol-PEG-thiol (MW of 3400; Laysan Bio), with acrylate and thiol mixed at a molar ratio of 1:1 for cross-linking. For sol–gel transition time characterization, 7.5 w/v% and 10 w/v% PEG hydrogel were respectively tested in PCR mix, LAMP mix, and culture media mix. PEG monomers were weighed to make 10× monomer solutions for PEG-acrylate and PEG-thiol separately. The weighed monomers were then dissolved either in water (Molecular Biology grade Water, Corning, Acton, MA, USA) for PCR and LAMP mix, or in TSB (BD Bacto Tryptic Soy Broth, Becton Dickinson and Company, Franklin Lakes, NJ, USA) for culture media mix. In addition to 2 μL of each 10× PEG monomer solution, for each 20 μL of reaction mix, the PCR mix contained 10 μL of ddPCR Supermix for Probes (BioRad, Hercules, CA, USA) and 6 μL of water; the LAMP mix contained 10 μL of 2× WarmStart LAMP Mastermix (New England Biolabs, Ipswich, MA, USA) and 6 μL of water; and the culture media mix contained 16 μL of TSB. The reaction mix was briefly vortexed. The sol–gel transition was considered started when lifting the pipet tip could draw filaments out of the reaction mix, and the transition was considered ended when the reaction mix formed a gelatinous lump.

### Development of the Disposable Droplet Generation Device

Each droplet generation device consisted of a 1.5 mL DNA LoBind tube (Eppendorf, Hamburg, Germany) and a blunt tip dispensing needle (LAOMA Amazon, Seattle, WA, USA) with the tip bent by a tweezer (VWR, Radnor, PA, USA). The tweezer and the needles were autoclaved (2540EP, Heidolph Brinkmann, Schwabach, Germany) prior to use. The oil phase was added to the bottom of the microcentrifuge tube, and the aqueous reaction mix was added to the Luer-lock of the needle. The device was then centrifuged (Centrifuge 5430R, Eppendorf) for 5 min. For optimization of droplet generation, fluorinated oil (HFE-7500 3 M Novec Engineering Fluid, 3M, Maplewood, MN, USA) supplied with 5% FluoroSurfactant (RAN Biotechnologies, Beverly, MA, USA) was added into the oil phase. The 20-μL aqueous phase contained 1 × WarmStart LAMP Mastermix and 50 μM calcein (Sigma-Aldrich, St. Louis, MO, USA). Four parameters including oil phase volume, needle inner diameter, centrifugal acceleration and oil volume added to the Luer-lock were investigated. Specific variables in details were as follows: (1) the oil phase volume of 40, 60, 80, and 100 μL, respectively, at the bottom of the tube in 34 Ga needles under 250*g* centrifugation; (2) needles of 30, 32, and 34 Ga (corresponding to inner diameter of around 160, 110, and 80 μm) under the condition of 250*g* centrifugation and 80 μL of oil phase volume; (3) the centrifugal accelerations of 50, 150, 250, 500, 1000*g* with 34 Ga needles and 80 μL of oil phase; and (4) additional oil phase added into the Luer-lock of 0, 10, and 20 μL in 34 Ga needles under 250*g* centrifugation with 80 μL of oil phase. Ten needles that generated droplets were randomly selected to measure the length of the bent tip by a ruler.

### Gelbead Generation and Thermal Stability Characterization

In all the following experiments, the device configuration was fixed with 34 Ga needles, 80 μL of oil phase, no additional oil at the Luer-lock, and 150*g* centrifugation run for 5 min. The droplet and Gelbead generation using the described device was respectively characterized with PCR mix, LAMP mix, and culture media mix. In each 20 μL of reaction mixture, the PCR mix contained 1× ddPCR Supermix and 50 μM calcein; the LAMP mix contained 1 × WarmStart LAMP Mastermix, and 50 μM calcein; the culture media mix was TSB with 1 mg/mL BSA (New England Biolabs) and 50 μM calcein. The mix was briefly pipet-mixed. The reaction mix for Gelbead generation contained 7.5 w/v% PEG hydrogel, added as 10× PEG monomers. For dispersion of PCR mix as droplets and Gelbeads, Droplet Generation Oil for Probes (BioRad) was used instead of fluorinated oil with 5% FluoroSurfactant.

For thermal stability characterizations, generated droplets or Gelbeads were extracted into PCR tubes (0.2 mL individual PCR tubes, BioRad) and incubated in a thermal cycler (T100, BioRad). The thermocycling protocol for PCR included 10 min of initiation at 95 °C, followed by 40 cycles of denaturation at 94 °C for 30 s, annealing at 52 °C for 60 s, and extension at 65 °C for 30 s. For LAMP heating, droplets or Gelbeads were incubated at 65 °C for 1 h.

### Bacterial Cell Culture and DNA Preparation

*Salmonellatyphi* (*S.typhi*, CVD 909), obtained from American Type Culture Collection (ATCC, Manassas, VA, USA), was employed as the model strain. *S.* Typhi was cultivated in TSB supplied with 1 mg/L of 2,3-dihydroxybenzoate (DHB, Sigma-Aldrich) in an incubator (Innova 42, New Brunswick Scientific, Edison, NJ, USA) shaking at 200 rpm at 35 °C for 14–16 h. The concentration of cultivated cells was estimated by OD 600 (NanoDrop 2000c Spectrophotometer, Thermo Scientific, Barrington, IL, USA). DNA was harvested using PureLink Genomic DNA Mini Kits (Fisher Scientific, Waltham, MA, USA) following the manufacturer’s instructions. For the single-cell encapsulation test, *Salmonella* Typhimurium GFP (ATCC 14028GFP) was cultivated in nutrient broth (Difco 23400, Becton Dickinson and Company) supplied with 100 μg/mL Ampicillin (Sigma-Aldrich) in an incubator shaking at 200 rpm at 37 °C for 14–16 h. The cell concentration was estimated by counting under a fluorescence microscope (Leica DMi8, Wetzlar, Germany).

### Gelbeads for Cell Viability Phenotyping

For single-cell encapsulation efficiency test, the cultivated *Salmonellatyphimurium* GFP (*S.typhimurium* GFP) was diluted 600 times for Gelbeads generation. The dilution factor was estimated from prior knowledge of harvested cell concentration and Gelbead volume. The number of cells encapsulated in each Gelbead was analyzed by fluorescence microscope imaging with a 20× objective. 79 Gelbeads were analyzed from 15 fluorescent images. For phenotyping experiments, 1 mL of overnight cultured *S.typhi* was freshly cultivated for 3 h in 5 mL of TSB supplied with 1 mg/L of DHB in an incubator shaking at 200 rpm at 35 °C. The cell concentration was verified to be around 0.135 by OD 600. AlamarBlue (Invitrogen, Carlsbad, CA, USA) was employed as the cell viability indicator. To address the fluctuation of excitation intensity and emission detection within a microscopic view, calcein was used as a reference dye. Each 20 μL of reaction mixture consisted of 1× AlamarBlue, 50 μM calcein, 1 mg/mL BSA, diluted *S.* Typhi cells, and the rest of the volume filled with DHB supplied TSB. 7.5 w/v% PEG hydrogel was added as 10× PEG monomers dissolved in DHB supplied TSB. After generation, the Gelbeads were incubated at 37 °C for 0–5 h. Gelbeads were extracted for imaging after 0, 1, 2, 3, and 4 h of incubation.

### Gelbead Digital PCR (gdPCR) Assay

The thermocycling protocol of gdPCR assay was the same as described in the Thermal Stability Characterization section. Each 20 μL of reaction mixture consisted of 1× ddPCR Supermix, 900 nM forward primer, 900 nM reverse primer, 250 nM probe, and 2 μL DNA sample or water. Additional 7.5 w/v% PEG hydrogel was added as 10× PEG monomers for gdPCR assays. The primers and probe were ordered from Integrated DNA Technologies (IDT, Coralville, IA, USA), with sequences (Supplementary Table S1) designed for specific detection of *S.typhi*, targeting a region in gene STY0201 for an amplicon size of 131 bp.^44^ For gdPCR optimization, the same DNA template concentration (600 times dilution from harvested) was added for gdPCR assays and ddPCR control. Optimal concentration of additional polymerase (OneTaq DNA polymerase, New England Biolabs) was investigated by supplying various concentrations to the described reaction mix incrementally at 0.025, 0.5, 0.1, and 0.2 U/reaction. For quantification assays, harvested DNA sample were serial diluted 100, 300, 600, 1500, and 24000 times for ddPCR and gdPCR. The reactions were prepared on iceblock (Carolina Chill Block, Burlington, NC, USA), and centrifugation temperature was set at 4 °C. Droplets or Gelbeads were generated in BioRad droplet generation oil, and were then extracted into PCR tubes for thermocycling. No-template controls were examined for each tested condition.

### Gelbead Digital LAMP (gdLAMP) Assay

The reagents for LAMP were acquired from New England BioLabs if not indicated otherwise. Each 20 μL of modified LAMP mix for digital single bacteria LAMP contained 1× isothermal buffer, 6 mM total MgSO_4_, 1.4 mM dNTP, 640 U/mL Bst 2.0 WarmStart polymerase, 1.6 μM FIB and BIP, 0.2 μM F3 and B3, 0.8 μM LF and LB, 1.5 mg/mL BSA, 1× LAMP dye.^49,50^ For gdLAMP assays, 7.5 w/v% PEG hydrogel was added as 10× PEG monomers. The primers, ordered from IDT with the sequences shown in Supplementary Table S1, were targeting a 196 bp region within the *S.typhi* specific gene STY1607.^51^ For gdLAMP and ddLAMP assays, harvested DNA was serial diluted 5, 20, 50, 100, and 200 times. The reactions were prepared on iceblock and centrifuged into 5% FluoroSurfactant supplied fluorinated oil at 4 °C. Droplets or Gelbeads were then extracted into PCR tubes for 30 min heating at 65 °C followed by 5 min polymerase deactivation at 80 °C. No-template controls were examined under the same protocol.

### Combined Phenotyping and gdPCR for Antibiotic Resistance Analysis

*S.typhi* cells were cultivated, encapsulated, and phenotyped following the same procedure as described in the section Gelbead Phenotyping. The phenotyped Gelbeads were subject to phase transfer and reagent infusion in preparation for in situ PCR. The chemical emulsion breaker was prepared by diluting 1*H*,1*H*,2*H*,2*H*-perfluorooctanol (PFO, Sigma-Aldrich) with HFE 7500 oil to make 20 vol % PFO stock. Excess oil below the Gelbeads was extracted and discarded. After 10 μL of PBS was added and briefly vortexed, 40 μL of 20 vol % PFO was added to the top, and the tube was mildly vortexed for 10 s. The mixture was then briefly centrifuged. All the liquids were drained with a pipet sticking to the bottom of the tube. Then 40 μL of water was added to the Gelbeads and the mixture was frozen at −20 °C for approximately 16 h. After thawing, the volume of the Gelbeads was roughly estimated by comparing the interface level of the total mixture and the pipet-removed water with the interface level of known volume. Concentrated PCR reagent mixture was added to the drained Gelbeads at twice their estimated volume. The concentrated PCR mixture was prepared 1.5 times the final component concentrations, which were similar to the recipe in gdPCR with doubled primers and probe concentration. The aqueous mixture of Gelbeads and PCR reagents was allowed to sit for 60 min. Gelbeads were then washed with oil for 3 times to eliminate remaining free aqueous phase. During each washing cycle, the mixture was pipet-mixed with additional 20 μL of BioRad droplet generation oil, and the fluids were pipet-drained. The washed Gelbeads were resuspended in 80 μL of BioRad droplet generation oil for PCR thermocycling. Before imaging, the Gelbeads were washed again with the oil to eliminate possible interference from the remaining aqueous droplets.

### Droplets and Gelbeads Imaging and Analysis

The droplets or Gelbeads to be analyzed were transferred into a viewing chamber made by adhering SecureSeal Hybridization Chamber (9 mm DIA × 1.0 mm Depth, Grace Bio-Laboratories, Bend, OR, USA) to a glass slide (VistaVision Microscope slides, VWR). The chambers were imaged under the fluorescence microscope using a 1.25× objective for droplets/Gelbeads generation, characterizations, and gdLAMP. For each sample in gdPCR and single cell phenotyping, five images of different area in the viewing chamber were taken using a 5× objective. Fluorescein isothiocyanate (FITC) filter was used, except for phenotyping experiments where Texas Red (TXR) filter was used in addition. In phenotyping experiments, the image data collected through TXR channel was normalized using the image data collected through FITC channel. For analysis of bright Gelbeads fraction, the data of each pixel was the intensity ratio of TXR channel to FITC channel. All images were analyzed using customized MATLAB scripts (Supplementary Files). For droplets and Gelbeads generation as well as thermal stability characterizations, the images were analyzed for individual compartment diameters. The diameters were further analyzed to calculate average compartment diameter and coefficient of variation (CV). For gdPCR, gdLAMP, and phenotyping assays, in addition to size analysis, the images were also analyzed for number of positive and negative compartments by setting a bright-dark threshold. Using the ratio of negative compartments to total compartments, the input DNA or cell concentrations were estimated by Poisson distribution.^52^ For images from phenotyping assays, since the distinction of dark and bright Gelbeads was hard to inspect visually, Gaussian fitting was used to advice the threshold (Figure S4).

## Supporting Information Available

MATLAB scripts for fluorescence image analysis and a python script for Monte Carlo cell distribution experiments (ZIP)
Video of simulated droplet generation with the developed needle-in-a-tube device (AVI)
PEG hydrogel cross-linking characterization data and sequences of primers and probe for PCR and LAMP assays, simulation results of varying parameters for droplet generation, setup and results of Monte Carlo simulation of cell distribution, example images of Gelbeads before incubation for cell phenotyping, MATLAB analysis and thresholding, calculated effective diffusivities, gdPCR and gdLAMP estimation compared with input DNA concentration, image of Gelbead aggregation observed in gdLAMP with the original assay recipe, combined phenotyping and in situ PCR, and fluorescence analysis of Gelbeads after in situ PCR, the results of PEG hydrogel cross-linking characterization, methods for droplet generation simulation, droplet generation performance and sources of error, droplet size based on simulation with varying physical parameters, methods and results of Monte Carlo simulation of cell distribution, the choice of microscope objective choices, challenges and solution in phase transfer and PCR reagent infusion for Gelbeads, and analysis of in situ PCR results (PDF)

## Supplementary Material

mt0c01615_si_001.zip

mt0c01615_si_002.avi
