# Supplementary material for: Single-Cell Phenotypic Analysis and Digital Molecular Detection Linkable by a Hydrogel Bead-Based Platform
Source: ACS Appl Bio Mater. 2021 Feb 12;4(3):2664–74. doi: 10.1021/acsabm.0c01615 (PMC7976597; doi:10.1021/acsabm.0c01615)
Supplement: Supplementary file 3 — mt0c01615_si_003.pdf [file mt0c01615_si_003.pdf]

Supplementary Information to

Single-cell phenotypic analysis and digital molecular detection linkable by a hydrogel bead-based platform

Yanzhe Zhu, Jing Li, Xingyu Lin, Xiao Huang and Michael R. Hoffmann*

Linde+Robinson Laboratories

California Institute of Technology

Pasadena, California 91125, USA

* Correspondence and requests for materials should be addressed to MRH

Tel: 626-395-4391 EM: mrh@caltech.edu

This PDF file includes:

Supplementary Tables S1 to S2

Supplementary Figures S1 to S9

Supplementary Notes S1 to S8

References

Supplementary Tables

Table S1.

<table>
<tr><th colspan="2"></th><th>LAMP mix</th><th>PCR mix</th><th>TSB media</th></tr>
<tr><td colspan="2">pH</td><td>8.8</td><td>8.3</td><td>7.3</td></tr>
<tr><td rowspan="3">7.5 w/v%</td><td rowspan="2">Sol-Gel transition time [min]</td><td>7.0</td><td>11.5</td><td>43.0</td></tr>
<tr><td>8.5</td><td>13.5</td><td>53.0</td></tr>
<tr><td>Pore size</td><td colspan="3">27nm</td></tr>
<tr><td rowspan="3">10 w/v%</td><td rowspan="2">Sol-Gel transition time [min]</td><td>4.5</td><td>8.5</td><td>18.0</td></tr>
<tr><td>5.5</td><td>10.0</td><td>23.5</td></tr>
<tr><td>Pore size</td><td colspan="3">25nm</td></tr>
</table>

Table S1. PEG hydrogel crosslinking characterization in bulk for LAMP mix, PCR mix, and TSB media. Sol-Gel transition time was experimentally determined (See **Online methods**). The pH values were supplied by the manufactures. The pore sizes were theoretically estimated for our gel concentration by scaling from experimentally measured mesh sizes, assuming a simplified hydrogel architecture.[1]

Table S2.

PCR	
Forward primer	5’ CGCGAAGTCAGAGTCGACATAG 3’
Reverse primer	5’ AAGACCTCAACGCCGATCAC 3’
Probe	5’ FAM AAGACCTCAACGCCGATCAC 3’

LAMP	
FIP	5’ AACTTGCTGCTGAAGAGTTGGACCGAATGACTCGACCATC 3’
BIP	5’ CCTGGGGCCAAATGGCATTATGCACTAAGTAAGGCTGG 3’
F3	5’ GACTTGCCTTTAAAAGATACCA 3’
B3	5’ AGAGTGCGTTTGAACACTT 3’
LF	5’ TCGGATGGCTTCGTTCCT 3’
LB	5’ CAAGGGTTTCAAGACTAAGTGGTTC 3’

Table S2. Sequences of primers and probe for PCR and LAMP assays. PCR primers and probes target a region in gene STY0201 specific for *S.* Typhi for an amplicon size of 131 bp.[2] LAMP primers target a 196 bp region within the *S.* Typhi specific gene STY1607.[3] The target regions for PCR and LAMP were both found to occur at one copy per cell, by searching the sequences within the complete genome of *S.* Typhi strain CT18 (Accession no. NC_003198) using Basic Local Alignment Search Tool (BLAST) [4].

Supplementary Figures

Figure S1.

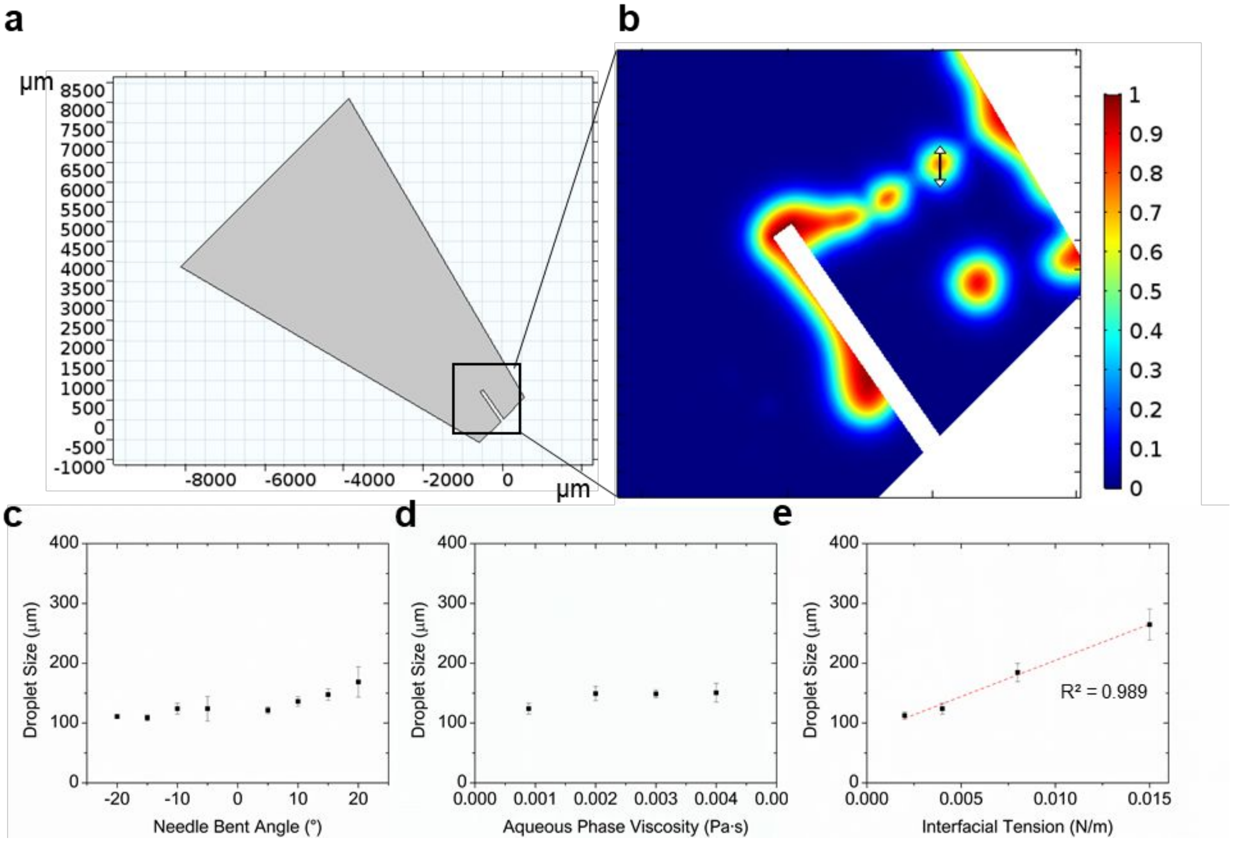

Figure S1. COMSOL simulation of needle-in-a-tube (NeaT) droplet generation. (a) Geometry of the fluid regime at the bottom of the microcentrifuge tube. (b) Example simulation result expressed in volume fraction of aqueous phase with zoomed-in view at near the needle tip where droplet pinch-off occurs. The droplet size was estimated by three diameter measurement of the droplet that just pinched off. The double-headed arrow represents an example diameter measurement. (c-e) Droplet sizes based on the simulation results of varying (c) needle bent angle, (d) aqueous phase viscosity, and (e) interfacial tension. Error bars represent standard deviation for three measurements.

Figure S2.

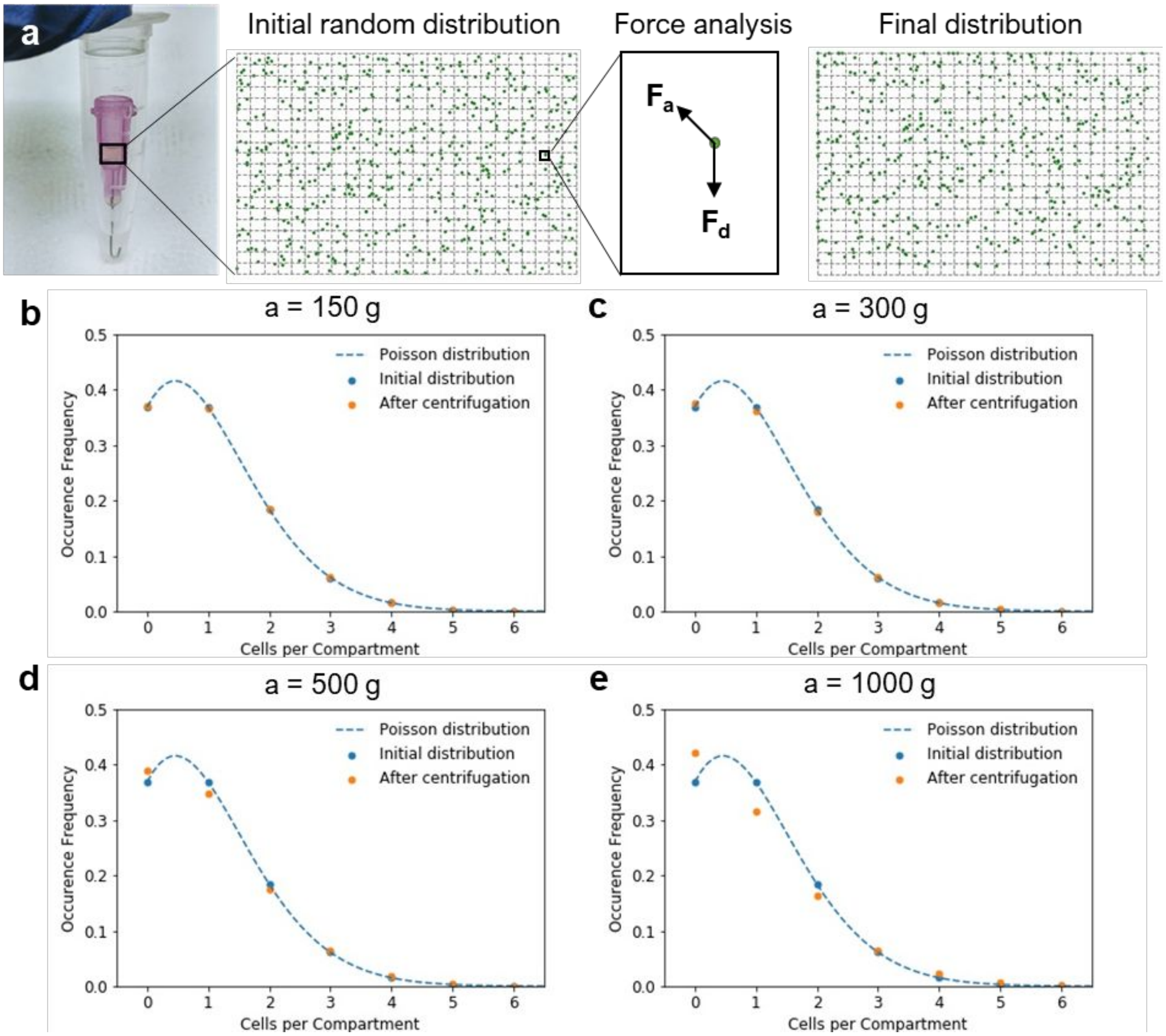

Figure S2. Setup and results of Monte-Carlo simulation of cell distribution during centrifugation. (**a**) The fluid regime inside the Luer-lock was approximated as a rectangular area where cells (represented by green dots) were initially distributed randomly. The final position of each particle after centrifugation was calculated based on the centrifugal force and the drag force exerted by the fluid. The dashed lines represent 200 μm × 200 μm virtual compartments. (**b-e**) The number of cell particles per compartment was counted and represented by occurrence frequency under centrifugal acceleration of (**b**) 150 g, (**c**) 300 g, (**d**) 500 g, (**e**) 1000 g.

Figure S3.

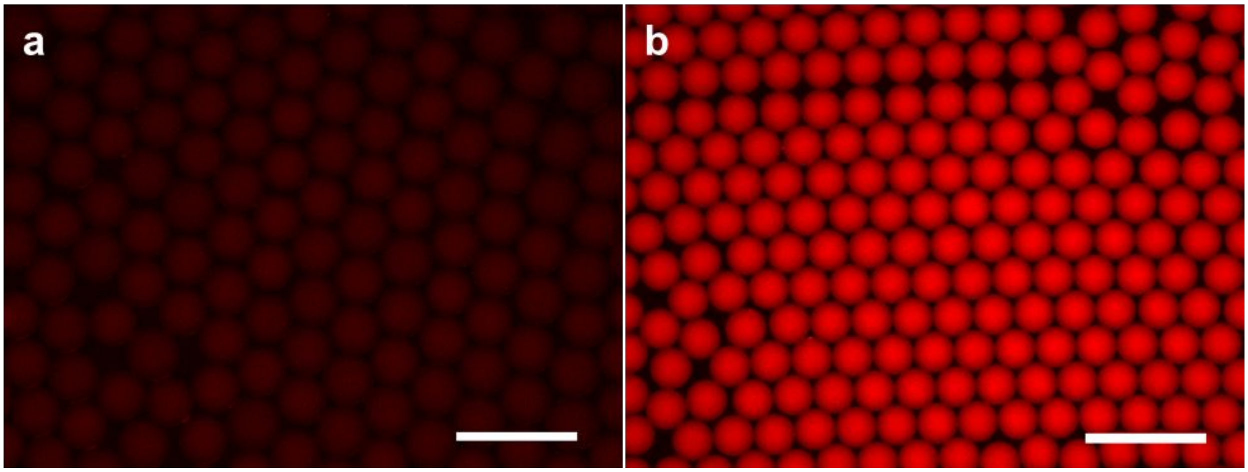

Figure S3. Example images of preliminary cell phenotyping experiments. With alamarBlue and media only and without incubation, Gelbeads appeared to be much brighter than (**a**) droplets even though (**b**) Gelbeads were imaged under a lower fluorescence (300 ms exposure for droplets and 25 ms exposure for Gelbeads). Scale bars, 500 μm.

Figure S4.

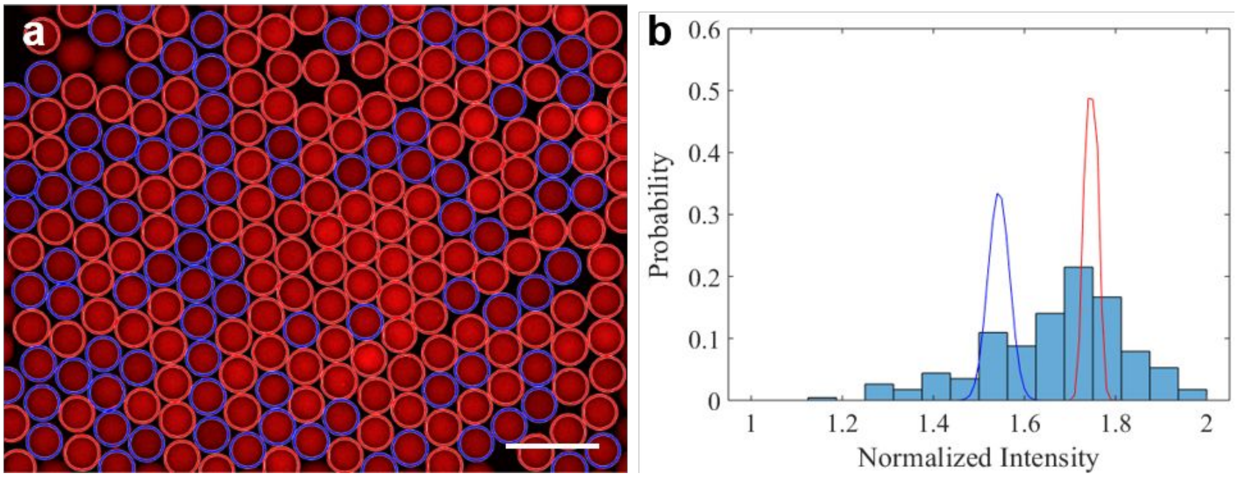

Figure S4. MATLAB analysis and threshold setting for images from phenotyping experiments. (**a**) An example image of phenotyping assay analyzed in MATLAB. Scale bar, 500 µm. The blue circles represent identified dark Gelbeads, and the red circles represent identified bright Gelbeads. (**b**) The histogram presents the occurrence probability of mean normalized intensity of Gelbeads analyzed based on the source image of (a). Gaussian fitting of the occurrence probability data generated two peaks, represented by the blue and red curves. The threshold suggested by this MATLAB script was set as the average of the mean (µ) of two peaks. This threshold was used to categorize negative and positive Gelbeads and produced the identification results on the left. We note that for 2 hours and 5 hours of incubation, the differences in normalized intensity of Gelbeads were too small for this thresholding method. In these cases, threshold enumeration and visual inspection were used instead for an approximately appropriate threshold.

Figure S5.

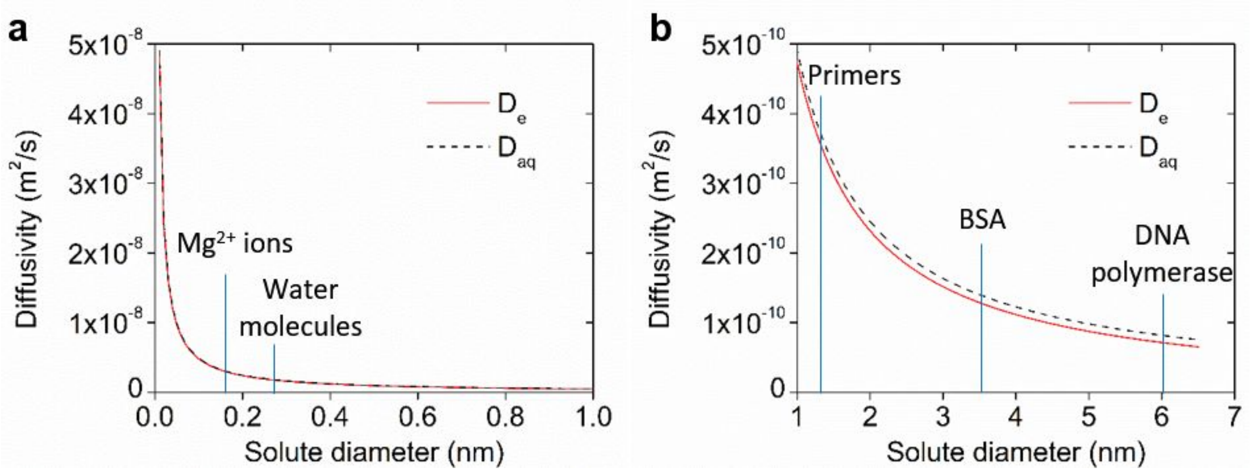

Figure S5. Calculated effective diffusivity (D_e) of solute in 7.5 w/v% PEG hydrogel matrix based on Weber et al.[5] and diffusivity in aqueous phase (D_{aq}) at 37 °C based on Stokes-Einstein equation as a function of solute hydrodynamic radius (**a**) from 0-1 nm and (**b**) from 1-7 nm. For functional molecules in the molecular assays approximate sizes reported in or calculated from literature [6-7] are indicated on the plots.

Figure S6.

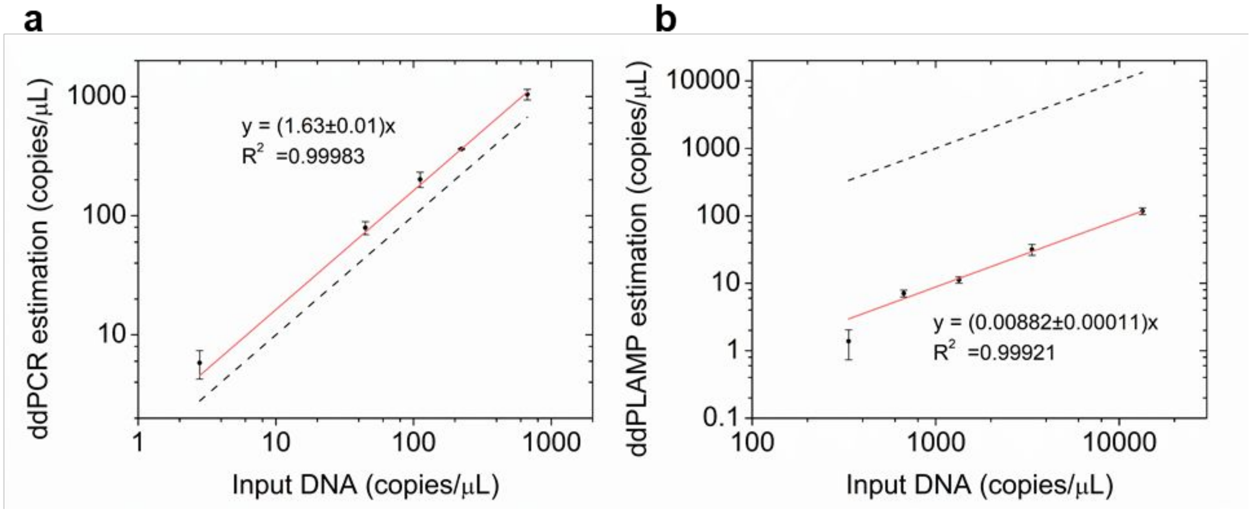

Figure S6. Estimated DNA concentration by (**a**) ddPCR for 24000, 1500, 600, 300, 100 times dilution of harvested *S.* Typhi DNA. and (**b**) ddLAMP for 200, 100, 50, 20, 5 times dilution of harvested *S.* Typhi DNA. compared with input DNA concentration. Input DNA concentration was calculated from dilution factor and OD600 measurement of cultured cells before DNA extraction using commercial kit. The dashed lines reference an exact match with input DNA concentration.

Figure S7.

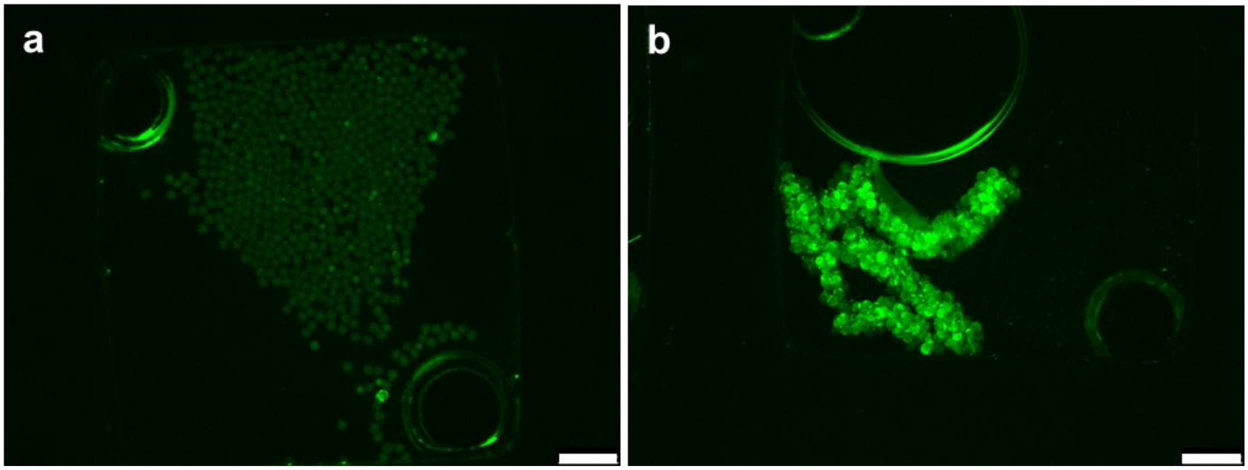

Figure S7. Gelbead aggregation was observed exclusively in positive samples during preliminary gdLAMP experiments. Example fluorescent images of Gelbeads with (**a**) no target template and (**b**) 100 times dilution of harvested *S*. Typhi DNA. Scale bars, 1 mm. For preliminary gdLAMP experiments, LAMP MasterMix was used (New England BioLabs) instead of the customizable LAMP recipe specified in **Online Methods**. Each 20 µL of reaction mix for contained 1× LAMP MasterMix, 1.6 µM FIB and BIP, 0.2 µM F3 and B3, 0.8 µM LF and LB, 1× LAMP dye. 7.5 w/v% PEG hydrogel was added as 10× PEG monomers. The heating protocol involved 65 °C for 30 min and then 80 °C for 5 min. Aggregation of Gelbeads observed for positive samples but not for no-template controls. The extent of aggregation indicates occurrence of severe crosstalking.

Figure S8.

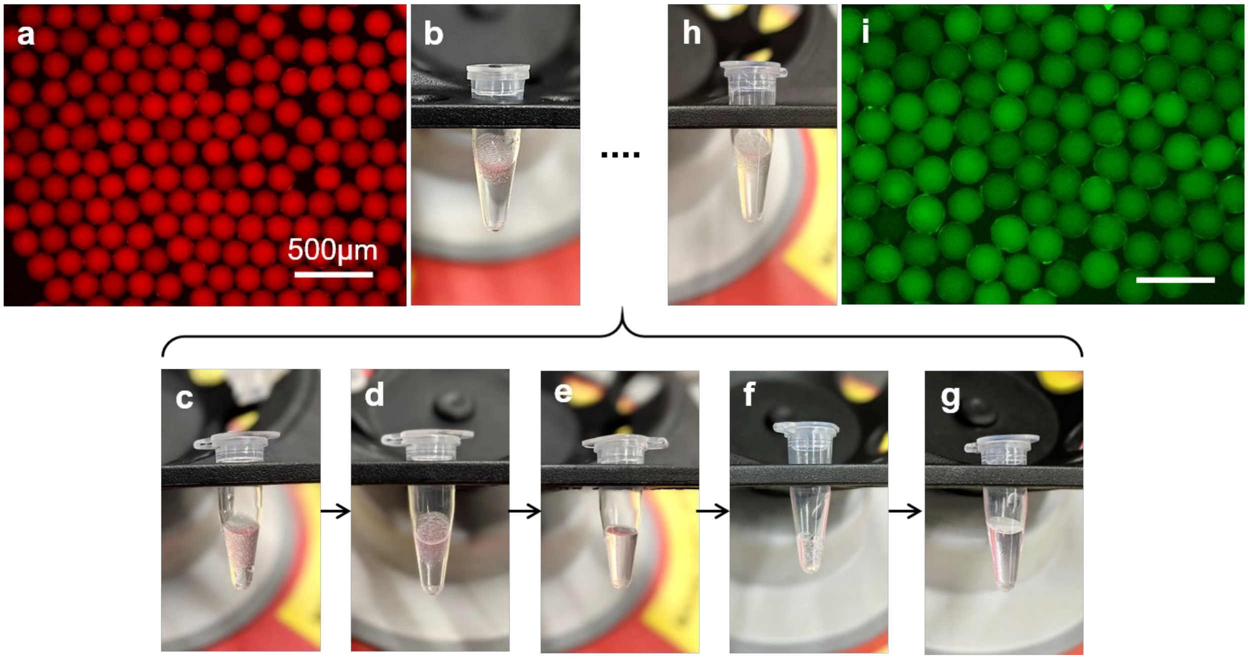

Figure S8. Combined phenotyping and *in situ* PCR. (**a**) Example fluorescence image of the Gelbeads after phenotyping. (**b**) The phenotyped Gelbeads were subject to reagent exchange, involving (**c**) breaking the emulsion with PFO, (**d**) facilitating phase separation by centrifugation, (**e**) removing all the liquid and adding water to freeze overnight, (**f**) draining the water with pipette, (**g**) incubating with concentrated PCR reagents, and (**h**) washing the Gelbeads with BioRad oil and resuspending for PCR. (**i**) Example fluorescence image of the Gelbeads that were further analyzed through *in situ* gdPCR. Scale bars, 500 µm.

Figure S9

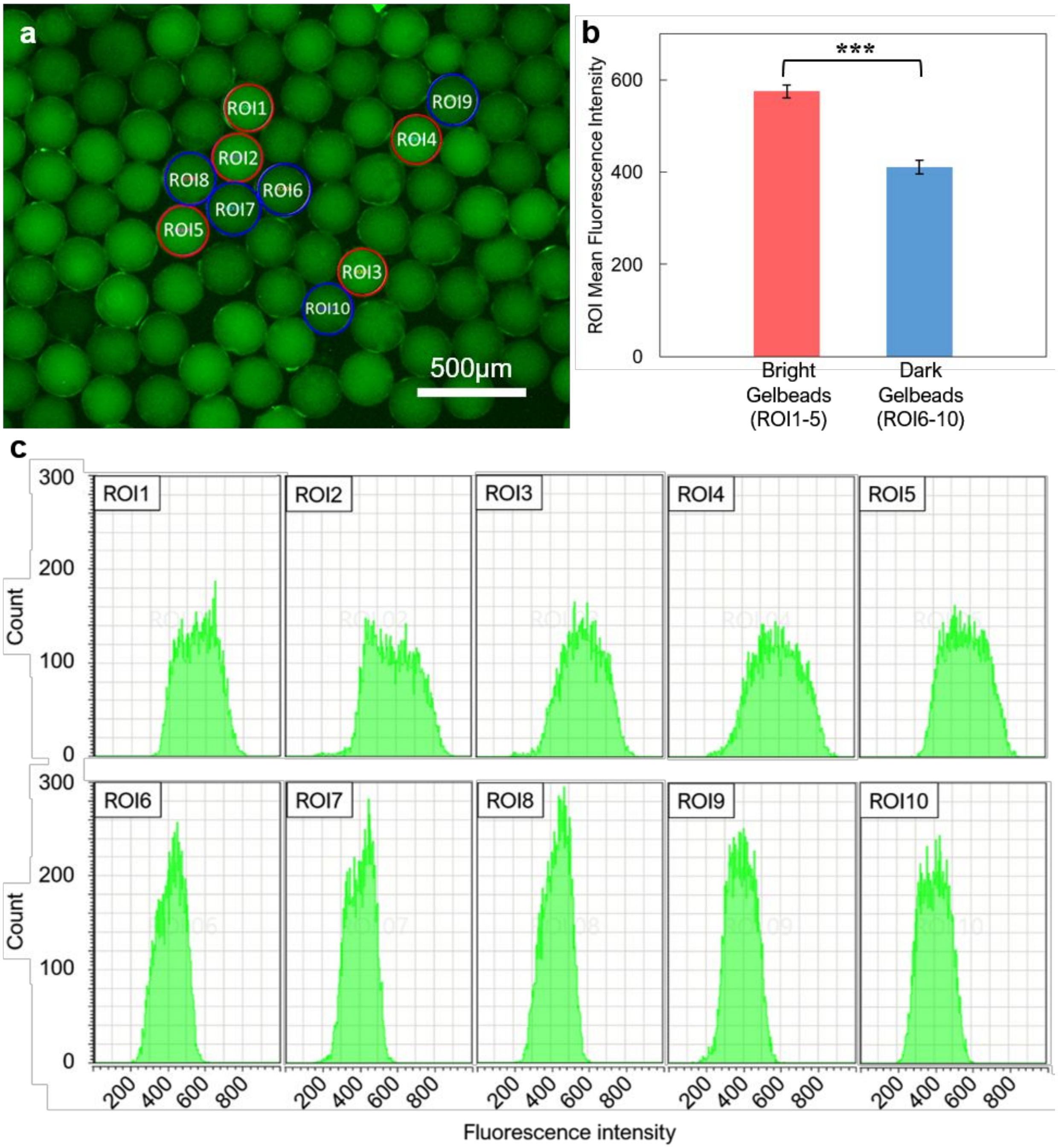

Figure S9. Fluorescence analysis of Gelbeads after *in situ* PCR. A fluorescence image was analyzed in the software (Leica Application Suite X) accompanied the fluorescence microscope.

(**a**) The fluorescence image with 10 bright and dark Gelbeads circled as region of interest (ROIs) to be analyzed. Bright Gelbeads (ROI1-5) are marked by red circles, and the dark ones (ROI6-10) are marked by blue circles. The ROIs were selected as pairwise neighboring bright and dark Gelbeads to limit the interference of the focus effect. Scale bar, 500 µm. (**b**) The mean fluorescence intensity within the ROIs were significantly different ($p < 0.001$, one-way ANOVA) for the analyzed bright and dark Gelbeads. The averaged mean fluorescence intensity of the bright Gelbeads was 40% higher than the dark ones. Error bars represent standard deviation for the 5 ROIs in each group. (**c**) Histograms of fluorescence intensity counts for the ROIs.

Supplementary Notes

Supplementary Note 1: Characterization of PEG hydrogel crosslinking

To identify the reasonable time frame for Gelbead generation, PEG gelation time in our targeted reaction matrices by bulk phase sol-gel transition experiments (online methods) was first measured. Ideally the compartmentalization process should be completed before the sol-gel transition starting, after which further crosslinking would considerably alter fluid properties such as viscosity and surface tension. For the three types of reaction matrix examined, results showed that the sol-gel transition start time spanned from 4.5 min to 43.0 min, and the crosslinking was accelerated by higher pH and higher monomer concentration (**Table S1**). Accordingly, the gelation time might be further extended by decreasing the crosslinking temperature [8]. We then estimated if the lower gel concentration considerably affects the hydrogel properties. The theoretical pore sizes for our crosslinked PEG were close for 7.5% and 10% gel (**Table S1**). Both would allow diffusion of functional molecules in our applications including water, ions, small DNA fragments, and proteins that size from below an angstrom to ~ 6 nm.[6] This estimation neglects non-ideality such as dangling ends or monomer self-interlinking, which might result in larger actual pore size.[1] Using the same PEG monomers, 10 w/v% hydrogel concentration has been utilized in cell encapsulation, multiple displacement amplification (MDA) and LAMP.[9-10] We reason that lowering the hydrogel concentration to 7.5 w/v% would benefit our applications by allowing more time for Gelbead generation and creating looser hydrogel network for reagent diffusion. Therefore, 7.5 w/v% PEG was used in further experiments.

Supplementary Note 2: Setup of the simulation model for centrifugal droplet generation

We performed finite element modeling using COMSOL Multiphysics[11] to investigate the droplet generation process at the needle tip. The fluid regime in the microcentrifuge tube outside the needle was modeled as a two-dimensional geometry, as illustrated in **Figure S1a**. The oil at the bottom of the tube was represented by the isosceles trapezoid, with the bottom width at 1.6 mm and the base angle at 75°. The needle tip, with a length of 1.8 mm and a diameter of 80 μm, was represented by the indent of the fluid regime. The needle bent angle, which is the angle between the axes of symmetry of the needle tip and the oil reservoir, was set at 10°. The whole fluid regime was tilted 45° to resemble the configuration during centrifugation.

The *laminar two-phase flow module* was used to solve the flow field for the aqueous and oil phases using level-set method. The aqueous phase was simulated with properties of water, with a density of 1×10^{3} kg□m^{-3} and a dynamic viscosity μ of 8.9×10^{-4} Pa□s. The oil phase was simulated with properties of the fluorinated oil HFE-7500, with a density of 1.614×10^{3} kg□m^{-3} and a dynamic viscosity μ of 1.243×10^{-3} Pa□s. The interfacial tension was set at 0.004 N□m^{-1}, based on measurement for water in HFE-7500 oil with fluorosurfactant[12]. The fluid regime was filled with pure oil phase initially. The inlet aqueous phase fluid was set as a laminar inflow with an entrance length of 1.8 mm and a velocity of 0.013 m/s, which was approximately calculated by the experimental rate of droplet generation. The fluid regime was subject to gravity in the -y direction and a centrifugal acceleration of 150 g in the -x direction. The time-dependent two-phase flow fields were simulated for 10 ms with steps of 0.05 ms.

The simulation in the supplementary video was generated using the default setting described above. Experiments were performed by varying needle bent angle (-20°, -15°, -10°, -5°, 5°, 10°, 15°, and 20°), aqueous phase viscosity (8.9×10^{-4} , 2×10^{-3} , 3×10^{-3} , and 4×10^{-3} Pa□s), and interfacial

tension (0.002, 0.004, 0.008, and 0.015 N□m^{-1}). Zoom-in surface plot of aqueous phase volume fraction (**Figure S1b**) was used to measure the droplet size at each condition. Three measurement of droplet diameter was taken for the droplet that just pinched off, and the standard deviation was calculated.

Supplementary Note 3: Droplet generation performance and sources of error

To achieve high throughput analysis of droplets with limited available instruments, we chose to analyze droplets through fluorescence imaging. As in the reported protocol, droplets were extracted into a viewing chamber, made by bonding a commercial plastic chamber onto a glass slide, for fluorescence imaging at 1.25× objective. We acknowledge that this protocol might have introduced systematic error in size characterizations by 1) pipetting droplets from the microcentrifuge tube into the viewing chamber, 2) noise difference from focus point to the edges within an image, and 3) image processing bias by MATLAB when identifying the circular-shaped edges. These sources of error would lead to overestimation of size distribution. Therefore, it is anticipated that the actual CV of the generated droplets or Gelbeads should be lower than reported. Generally, the reported sizes and CVs of generated droplets, optimized at 175 µm diameter with CV of 5%, are comparable to those generated by centrifugal microfluidics reported in literature. For example, Haeberle et al. used polymer-tube micronozzles for alginate bead generation, and reported beads generated at diameters tunable from 180 to 800 µm with CV of 7–16% [13]. As another example, using a lab-on-a-disk centrifugal droplet generation with, Schuler et al. reported droplet diameters of 120 to 170 µm with CV of 2–4% [14]. It should be noted that these values represent only 20 measurements of droplet under high microscope objective.

Supplementary Note 4: Effect of varying physical parameters on droplet generation

Through COMSOL simulation of the two-phase flow at the needle tip, the effect of the needle bent angle, aqueous phase viscosity, and interfacial tension on the size of the droplets generated is shown in **Figure S1c-e**, respectively. The needle bent angle, which is the angle between the stem and the bent tip, is shown to have limited impact on the droplet size (**Figure S1c**). When this angle is varied between ±10°, the droplet sizes were all around 126 μm within the standard deviations of each other. This theoretically corroborates that, despite the seemingly not rigorous manual fabrication, the trial-to-trial error of NeaT droplet generation may still be fairly small. Aqueous phase viscosity itself has negligible impact on droplet size interestingly, although it was observed in other droplet generation studies that the higher viscosity led to larger generated droplets.[15] We speculate that the viscosity effect may manifest by changing the fluid velocity, and therefore optimization is needed for a new system with different viscosity of phases. It is also observed that the droplet size increases with increasing interfacial tension linearly (**Figure S1e**, $R^2 = 0.989$). This relationship indicates that, if smaller droplet size is desired, it can be achieved by adjusting the type and concentration of the surfactant for a lower interfacial tension[12]. This result also suggests that optimization is needed for a new combination of aqueous and oil phases when extending to other applications. We note that the absolute value of the simulated droplet sizes might not be directly comparable to those measured experimentally, as the simulation was set up with a simplified 2D approximation of the system. For the experimental condition that involves biological reagents in the aqueous phase, the actual interfacial properties may deviate from the ideal values used. In summary, the results from the simulations indicate that the developed NeaT droplet generation method is theoretically unsusceptible to fluctuations caused by manual fabrication, and this method can be easily extended to other applications that requires water-in-oil droplets.

Supplementary Note 5: Numerical study of cell distribution under centrifugation

Monte Carlo experiments were carried out to investigate the patterns of particle compartmentalization before and after centrifugation. The fluid regime inside the Luer lock was represented by a 2D rectangular (5 mm × 4 mm) model, as shown in **Figure S2a**. The initial positions of the cell particles were stochastically generated. For each particle, after 5 minutes of centrifugation, the final position was estimated by the second equation of motion based on the drag force and centrifugal acceleration (**Figure S2a**), and was bounded by the rectangular regime. In each experiment, the number of particles inside each 200 μm × 200 μm square compartment was counted according to the initial and final cell positions, in order to approximate the partitioning of cells into droplet. The number of cell particles was set to 500 so that the average number of particles per compartment is close to our experimental condition (μ=1 cell/droplet). The fluid was simulated with properties of water, with a density of 1 × 10^3 kg□m^{-3} and a dynamic viscosity of 8.9 × 10^{-4} Pa□s. The particle was simulated with properties of gram-negative cells,[16] with a density of 1.1 × 10^3 kg□m^{-3} and a radius of 1 μm. The drag force was calculated from Stokes' Law:

$$F_d = 6\pi \cdot \eta \cdot R \cdot v$$

where η is the dynamic viscosity of water, R is the cell particle radius, and v is the flow velocity. The flow velocity was 3.4 × 10^{-6} m□s^{-1}, calculated based on the observation that the fluid was completely forced out of Luer-lock in 5 minutes. The effect of varying centrifugal acceleration (a) was studied. The result of each studied condition was averaged from the results of 500 runs. The python code ("celldist.py") is attached in supplementary files.

The Monte Carlo simulation results of final cell distribution among the compartments versus the original random distribution are compared for varying centrifugal acceleration of 150, 300, 500,

and 1000 g (**Figure S2b-e**). Our NeaT droplet generation uses a very low centrifugal acceleration of 150 g for optimal droplet monodispersity. The simulation results show that 150 g and even 300 g does not discernibly alter the cell compartmentalization pattern. It should be noted that, since the outflow from the Luer-lock was not considered, this simulation could overestimate the risk of crowding the cells and breaking the random distribution pattern through centrifugation. In the actual process, at most only a small fraction of the fluid-cell mixture is susceptible to the full 5 min of centrifugation. Therefore, these results suggest that the strength and the timespan of the centrifugation used in our NeaT droplet generation has negligible effect on altering the random cell distribution.

Supplementary Note 6: Microscope objective choice

For ddPCR and gdPCR, 5× objective was used in fluorescent microscope imaging to more accurately capture the assay quantification performance. Taqman probe, commonly used in PCR for enhanced detection specificity by hydrolysis upon encounter of specific sequence target, was employed in our PCR assay recipe. In this case, the fluorescence exhibited by negative compartments was too low to be distinguishable from the oil phase under 1.25× objective. To be able to count negative compartments, a zoomed-in view using 5× objective had to be used instead of 1.25× objective, which was employed to image ddLAMP and gdLAMP results. A commercial DNA-intercalating dye was used in LAMP so that the higher background fluorescence was observed in negative compartments. 1.25× objective could image an area of 0.785 cm^2 to include the whole viewing chamber at one image shot. The smaller view at 5× objective was compensated by taking 5 images of different areas in the viewing chamber. A similar strategy was applied in Gelbeads imaging for phenotyping experiments to better distinguish the Gelbeads with varying fluorescence levels.

Supplementary Note 7: Overcoming challenges of PCR reagent infusion for Gelbeads

Initial attempts on PCR reagent infusion for phenotyped Gelbeads failed to yield any bright Gelbead, despite the visually successful Gelbead phase transfer from oil to aqueous phase and then back to the oil phase. Hydrogel network has been reported to possibly form smaller pores on the side of higher interfacial tension [17]. It was thus suspected that, during Gelbead crosslinking in fluorinated oil, the aqueous-oil interface might have smaller pore sizes. The surface barrier then might have hindered the inward diffusion of essential PCR macromolecules when the Gelbeads were transferred into aqueous phase. To overcome the Gelbead surface barrier, we performed a freeze-thaw treatment on the phenotyped Gelbeads prior to reagent infusion. The overnight freezing of the Gelbeads in water was intended for expanding the pores through ice crystal formation, which has been utilized to fabricate macroporous hydrogel for biomedical applications [18]. After adding the freeze-thaw treatment while other protocols remained unchanged, the Gelbead *in situ* PCR showed successful amplification.

It was observed that the contrast between positive and negative Gelbeads was less visually apparent than in direct gdPCR (**Figure 5d-h**), likely due to enlarged pore size. In our experiments, the fluorescence intensity difference was distinguishable for positive and negative Gelbeads, but the larger pore size might cause potential problems. For example, the viability phenotyping assay with other fluorescence dyes, such as PrestoBlue, that are more soluble in fluorinated oil thus easier to cross the interfacial barrier might have more crosstalking. It was also observed that, after phase transfers even without freeze-thaw treatment, the diameter of the Gelbead increased approximately from 160 μm to 220 μm. Gelbead swelling during the phase transfer process should not affect the interpretation of downstream PCR results, since the positive or negative reading on a Gelbead only reveals the molecular information of its own encapsulated cells since phenotyping.

However, the actual volume of the swollen Gelbeads need to be cautiously estimated while preparing the concentrated PCR reagent mixture, so that the final component concentrations can be achieved accurately.

Supplementary Note 8: *In situ* PCR results

The fraction of positive Gelbeads was observed to be 43%, which falls below the theoretical value of 63%, the percentage of Gelbeads containing at least one *S.* Typhi cell. Systematic optimization of the recipe is needed to establish the quantitative accuracy of downstream *in situ* molecular analysis.

References

1. Raeber, G. P.; Lutolf, M. P.; Hubbell, J. A., Molecularly engineered PEG hydrogels: A novel model system for proteolytically mediated cell migration. *Biophys J* **2005,** *89* (2), 1374-1388.

2. Tran, V. T. N.; Karkey, A.; Dongol, S.; Hang, N. T.; Dunstan, S.; Holt, K.; Le, T. P. T.; Campbell, J. I.; Tran, T. C.; Nguyen, V. V. C.; Arjyal, A.; Koirala, S.; Basnyat, B.; Dolecek, C.; Farrar, J.; Baker, S., The sensitivity of real-time PCR amplification targeting invasive Salmonella serovars in biological specimens. *Bmc Infect Dis* **2010,** *10*.

3. Fan, F. X.; Yan, M. Y.; Du, P. C.; Chen, C.; Kan, B., Rapid and Sensitive Salmonella Typhi Detection in Blood and Fecal Samples Using Reverse Transcription Loop-Mediated Isothermal Amplification. *Foodborne Pathog Dis* **2015,** *12* (9), 778-786.

4. Coordinators, N. R., Database resources of the National Center for Biotechnology Information. *Nucleic Acids Res* **2018,** *46* (D1), D8-D13.

5. Weber, L. M.; Lopez, C. G.; Anseth, K. S., Effects of PEG hydrogel crosslinking density on protein diffusion and encapsulated islet survival and function. *J Biomed Mater Res A* **2009,** *90a* (3), 720-729.

6. Wu, Y. B.; Joseph, S.; Aluru, N. R., Effect of Cross-Linking on the Diffusion of Water, Ions, and Small Molecules in Hydrogels. *J Phys Chem B* **2009,** *113* (11), 3512-3520.

7. Fujimoto, B. S.; Miller, J. M.; Ribeiro, N. S.; Schurr, J. M., Effects of different cations on the hydrodynamic radius of DNA. *Biophys J* **1994,** *67* (1), 304-8.

8. Pritchard, C. D.; O'Shea, T. M.; Siegwart, D. J.; Calo, E.; Anderson, D. G.; Reynolds, F. M.; Thomas, J. A.; Slotkin, J. R.; Woodard, E. J.; Langer, R., An injectable thiol-acrylate poly(ethylene glycol) hydrogel for sustained release of methylprednisolone sodium succinate. *Biomaterials* **2011,** *32* (2), 587-597.

9. Xu, L.; Brito, I. L.; Alm, E. J.; Blainey, P. C., Virtual microfluidics for digital quantification and single-cell sequencing. *Nat Methods* **2016,** *13* (9), 759-62.

10. Huang, X.; Lin, X.; Urmann, K.; Li, L.; Xie, X.; Jiang, S.; Hoffmann, M. R., Smartphone-Based in-Gel Loop-Mediated Isothermal Amplification (gLAMP) System Enables Rapid Coliphage MS2 Quantification in Environmental Waters. *Environ Sci Technol* **2018,** *52* (11), 6399-6407.

11. COMSOL Multiphysics, The platform for physics-based modeling and simulation. *Retrieved from: http://www.comsol.com/comsol-multiphysics* **2015**.

12. Holt, D. J.; Payne, R. J.; Chow, W. Y.; Abell, C., Fluorosurfactants for microdroplets: interfacial tension analysis. *J Colloid Interface Sci* **2010,** *350* (1), 205-11.

13. Haeberle, S.; Naegele, L.; Burger, R.; Von Stetten, F.; Zengerle, R.; Ducree, J., Alginate bead fabrication and encapsulation of living cells under centrifugally induced artificial gravity conditions. *J Microencapsul* **2008,** *25* (4), 267-274.

14. Schuler, F.; Schwemmer, F.; Trotter, M.; Wadle, S.; Zengerle, R.; von Stetten, F.; Paust, N., Centrifugal step emulsification applied for absolute quantification of nucleic acids by digital droplet RPA. *Lab Chip* **2015,** *15* (13), 2759-2766.

15. Vladisavljević, G. T.; Kobayashi, I.; Nakajima, M., Effect of dispersed phase viscosity on maximum droplet generation frequency in microchannel emulsification using asymmetric straight-through channels. *Microfluidics and Nanofluidics* **2010,** *10* (6), 1199-1209.

16. Martinez-Salas, E.; Martin, J.; Vicente, M., Relationship of Escherichia coli density to growth rate and cell age. *Journal of bacteriology* **1981,** *147* (1), 97-100.

17. Savina, I. N.; Gun'Ko, V. M.; Turov, V. V.; Dainiak, M.; Phillips, G. J.; Galaev, I. Y.; Mikhalovsky, S. V., Porous structure and water state in cross-linked polymer and protein cryo-hydrogels. *Soft Matter* **2011,** *7* (9), 4276-4283.

18. Oxley, H.; Corkhill, P.; Fitton, J.; Tighe, B., Macroporous hydrogels for biomedical applications: methodology and morphology. *Biomaterials* **1993,** *14* (14), 1064-1072.
